# Missing Link in the Growth of Lead-Based Zintl Clusters: Isolation of the Dimeric Plumbaspherene [Cu_4_Pb_22_]^4–^

**DOI:** 10.1021/jacs.1c10106

**Published:** 2022-04-22

**Authors:** Harry W. T. Morgan, Cong-Cong Shu, Zhong-Ming Sun, John E. McGrady

**Affiliations:** † Department of Chemistry, University of Oxford, South Parks Road, Oxford OX1 3QR, U.K.; ‡ State Key Laboratory of Elemento-Organic Chemistry, Tianjin Key Lab of Rare Earth Materials and Applications, School of Material Science and Engineering, Nankai University, Tianjin 300350, China

## Abstract

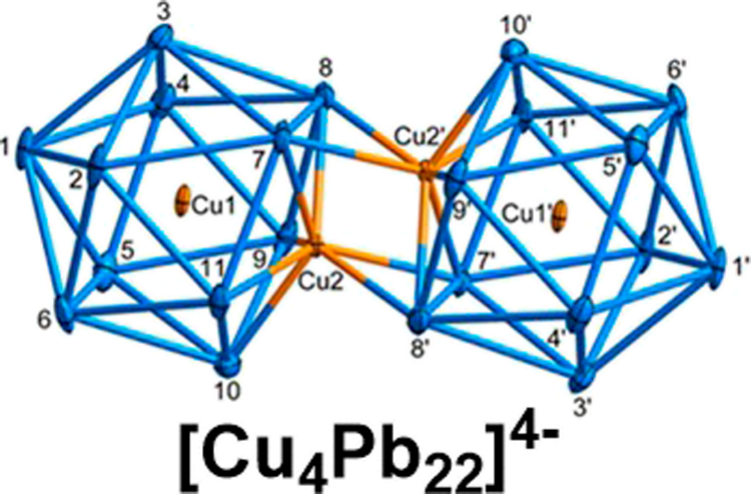

We report here the structure of an endohedral plumbaspherene, [Cu_4_Pb_22_]^4–^, the gold analogue of which was previously postulated to be a “missing link” in the growth of larger clusters containing three and four icosahedral subunits. The cluster contains two [Cu_2_Pb_11_]^2–^ subunits linked through a Cu_2_Pb_4_ trigonal antiprism. Density functional theory reveals that the striking ability of mixed Pb/coinage metal Zintl clusters to oligomerize and, in the case of Au, to act as a site of nucleation for additional metal atoms, is a direct consequence of their *n*d^10^(*n* + 1)s^0^ configuration, which generates both a low-lying (*n* + 1)s-based LUMO and also a high-lying Pb-centered HOMO. Cluster growth and nucleation is then driven by this amphoteric character, allowing the clusters to form donor–acceptor interactions between adjacent icosahedral units or to additional metal atoms.

## Introduction

The chemistry of Zintl ions, and in particular those containing endohedral transition metals, has seen a rapid expansion in recent years.^1−5^ Much of this has been driven by an innate interest in the nature of the chemical bond in these typically highly symmetric molecules, but it is becoming increasingly apparent that they are much more than mere ornaments. Potential applications in materials science have been highlighted in the recent literature,^6^ and the use of Zintl ions in catalysis is also beginning to be explored; recent examples include the catalysis of the reverse water gas shift reaction^7^ and the hydrogenation of alkenes.^8^ It is not always clear whether the Zintl cluster retains its structure throughout the course of the catalytic cycle, but nevertheless the presence of transition and main-group metals in a controlled ratio may play an important role in controlling reactivity. The key to realizing the full potential of Zintl ions in catalysis or in materials science will necessarily lie in the development of rational synthetic routes to generate ever larger clusters with tailored structures and elemental compositions.

**Figure 1:**
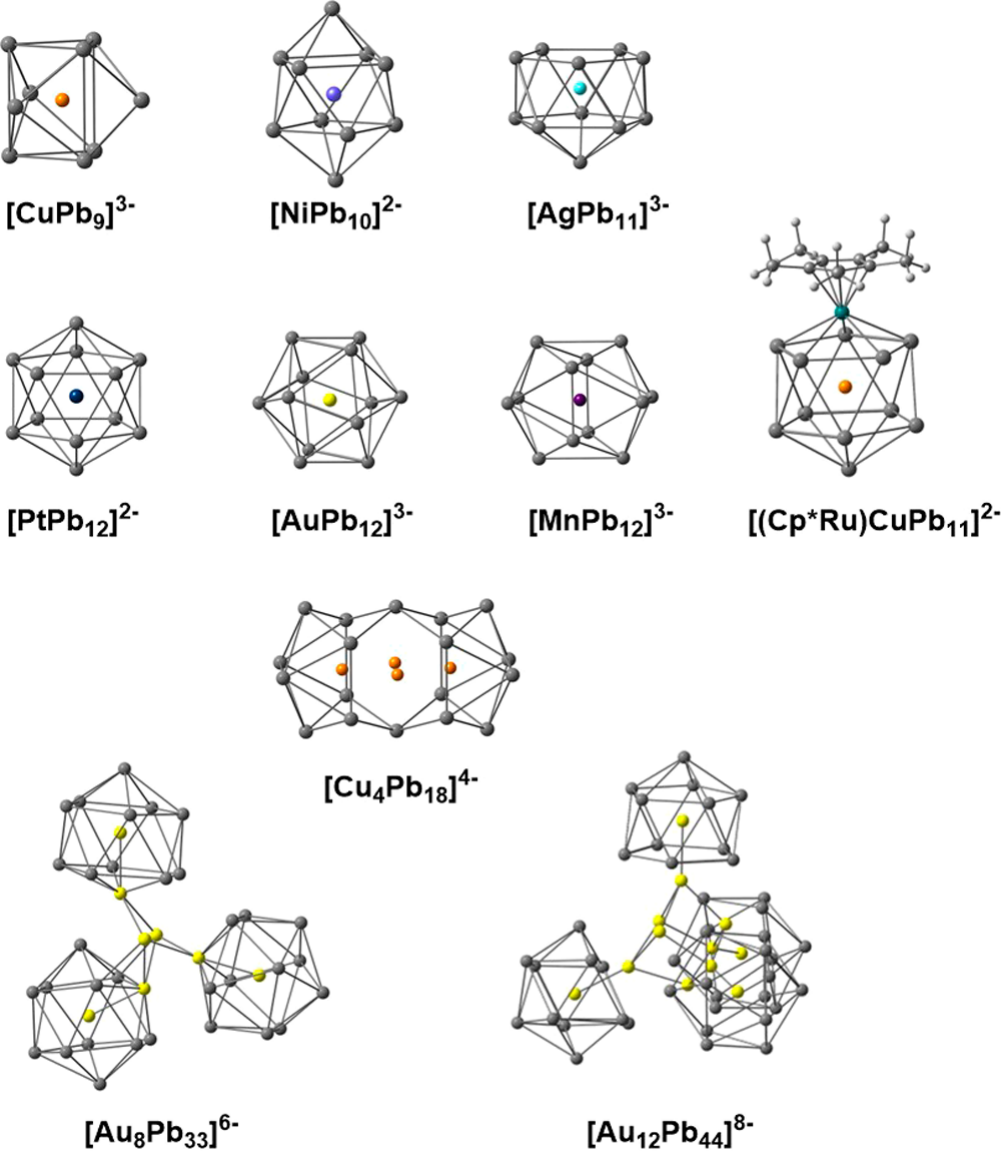
Structures of crystallographically characterized Pb-based Zintl ions.

Typical synthetic protocols used in contemporary work involve the combination of a main-group metal cluster (such as the Pb_9_^4–^ precursor used in this paper) with a source of low-valent transition metal ions, at high temperatures and in the presence of large counter-ions. The crystalline products are often highly sensitive to both air and moisture, but nevertheless a now extensive family of clusters with stoichiometries MPb_9–12_ has been synthesized in this way, including [CuPb_9_]^3–^,^9^ [NiPb_10_]^2–^,^10^ [AgPb_11_]^3–^,^11^ [MPb_12_]^*n*−^ (M = Au,^12^ Ni, Pd, Pt,^13^ Co, Rh, Ir,^14^ Mn^15^), and [(Cp*Ru)CuPb_11_]^2–^^16^ (Figure 1). Our understanding of the mechanism of growth of these clusters from smaller component parts remains limited, although a small number of recent studies have begun to address this critical issue using a combination of X-ray crystallography, mass spectrometry, and computational analysis.^17,18^ In the formation of the group-5 metal clusters [TaGe_8_As_4_]^3–^ and [TaGe_8_As_6_]^3–^, for example, fusion of the known tetrahedral [Ge_2_As_2_]^2–^ unit with the (unknown) Ta-containing fragments [TaGe_3_]^−^ and [TaGe_4_As_2_]^−^ has been shown to provide a viable route to the isolated products.^17^

The growth of even larger clusters containing multiple deltahedral units and/or transition metals, which offers the potential for a much wider range of M/E ratios along with the possibility of metal–metal bonding, presents a substantial synthetic challenge simply because the individual building blocks such as the ones shown in Figure 1 typically carry high negative charges. Nevertheless, the linking of discrete deltahedra through covalent bonds has been used to great effect in the oxidative coupling of Ge_9_^4–^, which can yield oligomers, polymers, and a mesoporous germanium phase.^19^ A recent report has also suggested that a centered [CoGe_9_]^5–^ unit can fuse to generate a condensed [Co_2_Ge_17_]^6–^ cluster where two Ge_9_ units share a common vertex.^20^ This structural motif is in fact relatively common in Zintl-ion chemistry,^21,22^ as are others where the component deltahedra are fused *via* edges or even hexagonal faces,^23−25^ although details of their formation mechanisms remain elusive. Transition metal ions can also be used to link distinct cluster units while also buffering some of the negative charge, as for example in [Ge_9_MGe_9_]^*q*−^ (M = Cu, Zn, In),^26−28^ [Ge_9_ZnGe_9_ZnGe_9_]^10–^,^29^ [Ge_9_HgGe_9_HgGe_9_HgGe_9_]^10–^,^30^ and polymeric [MGe_9_]_∞_^2–^ (M = Zn, Hg).^31,32^ In a small number of cases, metal dimer or trimer units have been used as the linker, as for example in [Pb_9_Cd-CdPb_9_]^6–^,^33^ [Ge_9_M-MGe_9_]^6–^ (M = Zn, Cd),^29^ and [Ge_9_Au_3_Ge_9_]^5–^.^34^

In a recent publication, we reported the synthesis and structures of a series of mixed Au/Pb clusters including [Au_8_Pb_33_]^6–^ and [Au_12_Pb_44_]^8–^, both of which contain icosahedral Au_2_Pb_11_ units arranged around a central Au_2_ or Au_4_ core, respectively.^11^ These new structures highlight the prominent role of the centered icosahedron as a fundamental unit in cluster growth, particularly in the heavier tetrels. Given that all of the clusters in Figure 1 have been isolated, it is perhaps surprising that the coalescence or fusion of endohedral units to form larger clusters is not a more common observation. The Coulombic barrier to fusion of highly charged components probably plays a part in this, but the isolation of [Au_8_Pb_33_]^6–^ and [Au_12_Pb_44_]^8–^ shows that this barrier is not insurmountable. We have proposed that in these mixed Au/Pb clusters, the inter-icosahedral bonding arises from strong donor–acceptor interactions between the Pb-based HOMO of one unit and the 6s orbital of the surface Au on another.^11^ Given the proven existence of clusters containing three ([Au_8_Pb_33_]^6–^ and [Au_12_Pb_44_]^8–^) icosahedral units, we also proposed that the coalescence of two such Au_2_Pb_11_ icosahedra to form dimeric [Au_4_Pb_22_]^4–^ might play a significant role in the initial stages of the reaction, but we were never able to isolate this or any other dimeric species, leaving a frustrating missing link between the isolated icosahedra and the condensed clusters. In this paper, we report the isolation and structural characterization of the copper analogue, [Cu_4_Pb_22_]^4–^, an observation that establishes at least that this motif is viable for mixed coinage metal/lead clusters. We then use this new information to propose an all-encompassing cluster-growth pathway that is supported by detailed calculations performed with density functional theory. These calculations establish the place of the new [M_4_Pb_22_]^4–^architecture in the context of the known chemistry of the Cu/Pb, Ag/Pb, and Au/Pb families.

## Experimental Methods

### Synthesis

All manipulations and reactions were performed under a nitrogen atmosphere using standard glovebox techniques. K_4_Pb_9_ was synthesized by heating a stoichiometric mixture of the elements at 850 °C for 36 h in a sealed niobium tube. [CuMes(PPh_3_)_2_] (Mes = 2,4,6-trimethylbenzyl) was synthesized according to literature procedures.^35^ 4,7,13,16,21,24-Hexaoxa-1,10-diazabicyclo[8.8.8]hexacosane ([2.2.2]-crypt, Sigma-Aldrich 98%) was dried under vacuum for several hours and transferred to a glovebox for use. Toluene (Aldrich, 99.8%) was distilled over sodium in a nitrogen atmosphere and also stored in a glovebox prior to use. Ethylenediamine (en) (Aldrich, 99%) and dimethylformamide (DMF) (Aldrich, 99.8%) were freshly distilled over CaH_2_ prior to use. 120 mg (0.059 mmol) of K_4_Pb_9_ was dissolved in 3 mL DMF along with 88 mg (0.236 mmol) of 2,2,2-crypt and 41 mg (0.059 mmol) of [CuMes(PPh_3_)_2_]. The resultant brown solution was stirred for 3 h at room temperature and then filtered with glass wool and the filtrate layered with 4 mL toluene. After 7 days, black block-like crystals of [K(2.2.2-crypt)]_4_[Cu_4_Pb_22_] were obtained in 25% yield (based on the amount of Pb present).

### Crystallography

Crystallographic data were collected on a Rigaku XtalAB Pro MM007 DW diffractometer with graphite monochromated Cu Kα radiation (λ = 1.54184 Å). Structures were solved using direct methods and then refined using SHELXL-2014 and Olex2 to convergence,^36,37^ where all the non-hydrogen atoms were refined anisotropically. All hydrogen atoms of organic groups were placed using geometrical considerations (CCDC reference 2054778). Full details of the crystallography are given in the Supporting Information, Table S1.

### Energy Dispersive X-ray Spectroscopy

Energy Dispersive X-ray spectroscopy was performed using a scanning electron microscope (Hitachi S-4800) equipped with a Bruker AXS XFlash detector 4010. Data acquisition was performed with an acceleration voltage of 20 kV and an accumulation time of 150 s.

### Electrospray Ionization Mass Spectrometry

Negative ion mode ESI-MS of the DMF solutions of a single crystal of [Cu_4_Pb_22_]^4–^ were measured on an LTQ linear ion trap spectrometer from Agilent Technologies, ESI-TOF-MS (6230). The spray voltage was 5.48 kV, and the capillary temperature was maintained at 300 °C. The capillary voltage was 30 V. The samples were made up inside a glovebox under a nitrogen atmosphere and rapidly transferred to the spectrometer in an airtight syringe by direct infusion with a Harvard syringe pump at 0.2 mL/min.

### Computational Methods

All DFT calculations were performed using the Amsterdam density functional (ADF) package, version 2019.304.^38^ Slater-type basis sets of triple-zeta + polarization quality were used on all atoms, with orbitals up to 2p (Cu), 3d (Ag), and 4d (Au, Pb) included in the frozen core.^39^ The Perdew–Becke–Ernzerhof (PBE)^40^ functional was used in all calculations, which were spin-restricted throughout. Relativistic effects were incorporated using the zeroth-order relativistic approximation (ZORA).^41^ The confining effect of the cation lattice was approximated using a continuum solvent model with a dielectric constant of 78.39.^42^ Open-shell systems are computed using spin-unrestricted DFT at the same level of theory. Fragment calculations were also performed with the same functional, basis sets, and solvation model, according to the extended transition state approach of Ziegler and Rauk.^43^ All stationary points were confirmed to be minima or transition states by the presence of none or one imaginary vibrational frequency, respectively. In some case, additional small imaginary frequencies (<10i cm^–1^) were found using the analytical frequencies module in ADF, but subsequent rescanning of these modes using numerical differentiation and a small step size (disrad = 0.002) showed these to be small and real.

## Results and Discussion

### Patterns of Cluster Growth

There exists an already substantial body of experimental evidence in the literature that we can use as a framework to build a model of cluster growth for mixed lead/coinage metal atoms, including

1. Structural characterization of tricapped trigonal prismatic [CuPb_9_]^3–^ and (distorted) icosahedral [AuPb_12_]^3–^.^9,12^ Both clusters have the skeletal electron count of 2*n* + 4 typically associated with a *nido* geometry, yet they retain the highly symmetric (albeit somewhat distorted) structures more commonly associated with a *closo* count of 2*n* + 2. The preference for these highly symmetric structures is probably driven by the spherically symmetric potential imposed by the endohedral metal, which in turn favors an approximately spherical arrangement of atoms over inherently less spherical *nido* alternatives.
2. Structural characterization of the approximately *C*_5*v*_-symmetric nido-[AgPb_11_]^3–^ cluster.^11^ This also has a skeletal electron count of 2*n* + 4, and it does adopt a classically *nido* geometry with one open face. The contrast in behavior to [CuPb_9_]^3–^ and [AuPb_12_]^3–^ may simply reflect the fact that there is no high-symmetry structure available for an 11-vertex cluster, so the distinction between *closo* and *nido* is less sharp than in the 12-vertex analogues.
3. Electrospray ionization mass spectrometry (ESI-MS) data that confirms the presence of clusters with stoichiometry MPb_11_ for both M = Ag and Au,^11^ M_2_Pb_9_ for M = Cu,^44^ and M_2_Pb_11_ for M = Au.^11^
4. Structural characterization of *D*_2*h*_-symmetric [Cu_4_Pb_18_]^4–^. This cluster is formed under very similar conditions to the title compound, [Cu_4_Pb_22_]^4–^, the only significant difference being the source of low-valent copper: [CuMes(PPh_3_)_2_] (Mes = mesityl) for [Cu_4_Pb_22_]^4–^ but [Cu_4_Mes_4_(THT)_2_] (THT = tetrahydrothiophene) for [Cu_4_Pb_18_]^4–^.^44^
5. Structural characterization of larger clusters [Au_8_Pb_33_]^6–^ and [Au_8_Pb_44_]^8–^ based on the fusion of approximately icosahedral Au_2_Pb_11_ units with two or four additional metal atoms.^11^ The presence of zerovalent metal atoms in these clusters means that they can be considered as models for the earliest stages of the nucleation of Au nanoparticles.

To this body of data, we now add a new structure, that of [Cu_4_Pb_22_]^4–^.

### Synthesis and X-ray Structure of the [Cu_4_Pb_22_]^4–^ Anion

**Figure 2:**
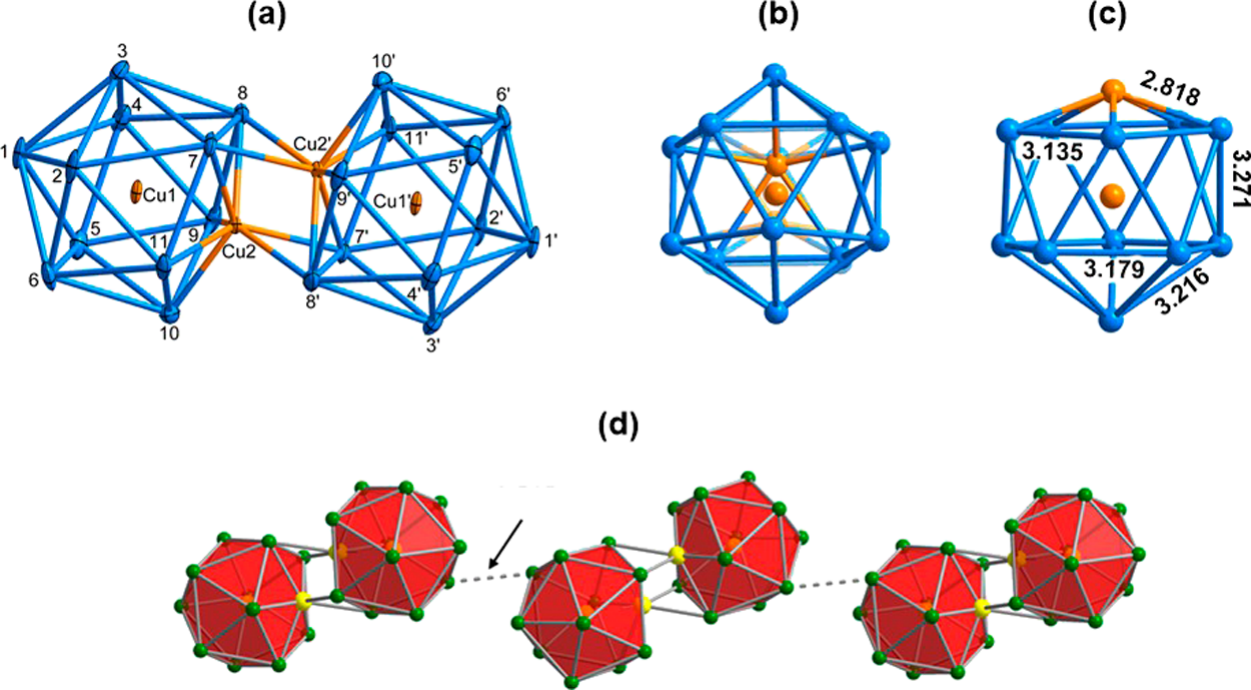
(a,b) Structure of the [Cu_4_Pb_22_]^4–^ anion viewed along two orthogonal axes. Thermal ellipsoids are set at the 70% probability level; (c) structure of one [Cu_2_Pb_11_]^2–^ subunit: bond lengths are the average of symmetry-related Cu–Pb and Pb–Pb distances, in Å; (d) structure of the one-dimensional chain of [Cu_4_Pb_22_]^4–^.

K([2.2.2-crypt)]_4_[Cu_4_Pb_22_] (1) was formed in the reaction of K_4_Pb_9_ and the organometallic compound [CuMes(PPh_3_)_2_] in ethylenediamine in the presence of [2.2.2-crypt] and crystallized in the monoclinic space group *C*2/*c*. Our attempts to isolate silver and gold analogues of 1 using [(AgMes)_4_] and [Au(Mes) (PPh_3_)] as sources of the coinage metal, respectively, have, unfortunately, not yielded crystals of sufficient quality to perform single crystal X-ray diffraction experiments. The [Cu_4_Pb_22_]^4–^ unit in 1 is an ellipsoidal plumbaspherene derivative with approximate *C*_2*h*_ symmetry (Figure 2a). The cluster contains two distinct Cu_2_Pb_11_ icosahedral units, which bind in a face-to-face manner *via* two CuPb_2_ triangles. The central region of the cluster is a distorted Cu_2_Pb_4_ trigonal antiprism with a Cu2–Cu2′ distance of 2.502(4) Å, only slightly shorter than those within the icosahedra (Cu1–Cu2, 2.508(3) Å). The average Cu–Pb bond length of 3.031 Å to the endohedral Cu atoms (Cu1, Cu1′) is very similar that in the Cu-centered cluster [CuCp*RuPb_11_]^3–^^16^ while distances to the apical copper atoms (Cu2, Cu2′) are somewhat shorter, at 2.8184 Å, and much shorter than the Pb–Pb distances within the icosahedra, which are within the normal range (3.214 Å).^11,12^ The cumulative effect of these differences in Cu–Pb and Pb–Pb distances is that the individual [Cu_2_Pb_11_]^2–^ icosahedra are strongly compressed along the Cu–Cu axis. The Cu_2_Pb_4_ antiprism at the center of the dimer unit has four short Cu–Pb contacts (Cu2-Pb7′, Cu2′-Pb7 = 2.8642(19) Å and Cu2-Pb8′, Cu2′-Pb8 = 2.925 Å), almost as short, in fact, as the Cu–Pb bonds within the icosahedra themselves. Similar trigonal antiprismatic Cu_2_Ge_4_ and Cu_2_Sn_4_ motifs have been reported previously in clusters containing either two 10-vertex or two 9-vertex deltahedra ([Cu_2_Ge_18_Mes_2_]^4–^, Cu–Cu = 2.5214(7) Å and [Cu_2_Sn_10_Sb_6_]^4–^, Cu–Cu = 2.563(3) Å, respectively).^45,46^ The dimeric nature of these three clusters offers an alternative formulation, as [(Cu_2_Pb_11_)_2_]^4–^, [(CuGe_9_Mes)_2_]^4–^, and [(CuSn_5_Sb_3_)_2_]^4–^, adopted by Dehnen and co-workers in their report of the latter, where they also concluded that no Cu–Cu bond was present despite the short Cu–Cu distance. We have previously made the same point in the case of [Cu_4_Pb_18_]^4–^^44^ (Cu–Cu = 2.56 Å av), and so the structural evidence presented here suggests that integrity of the [Cu_4_Pb_22_]^4–^ cluster is also maintained by the Cu–Pb and Pb–Pb interactions rather than any direct Cu–Cu covalent bonding. There are also additional secondary Pb–Pb interactions between Pb atoms of the two icosahedra at 3.5345(8) Å (Pb8–Pb11′ and Pb11–Pb8′) and 3.5695(8) Å (Pb7–Pb9′ and Pb9–Pb7′). Finally, adjacent [Cu_4_Pb_22_]^4–^ clusters are linked by Pb–Pb bonds of very similar length (3.5153 (11) Å) to form a one-dimensional chain (Figure 2d).

**Figure 3:**
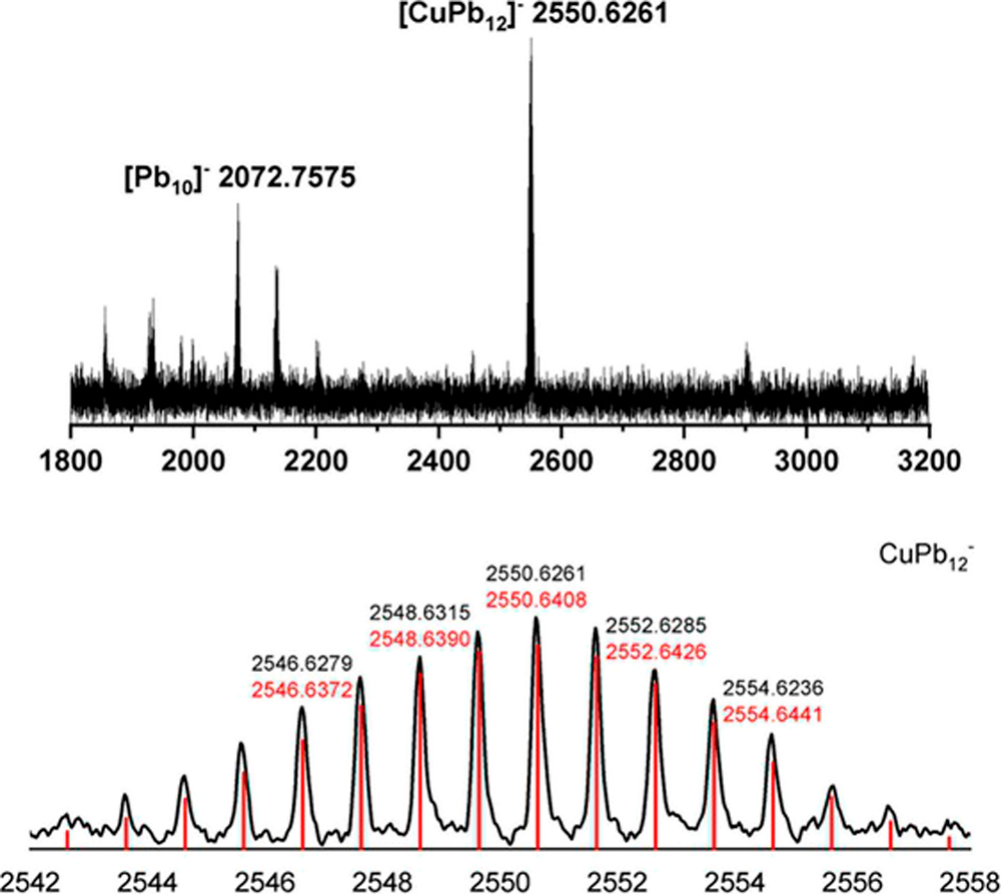
ESI-MS of a solution of 1 in DMF.

The ESI-MS of a solution made up by dissolving a single crystal of 1 in DMF is shown in Figure 3. A peak corresponding to the parent ion, [Cu_4_Pb_22_]^*z*−^, is absent from the spectrum, probably reflecting the facile fragmentation of the dimer into smaller icosahedral components. The most intense peak in the spectrum is not, however, due to the fragmentation product [Cu_2_Pb_11_]^−^, but rather to [CuPb_12_]^−^ at *m*/*z* 2550.6. If the Cu^+^ ion is endohedrally encapsulated, this [CuPb_12_]^−^ cluster has a *closo* electron count of 50 and is isoelectronic with a number of isolated icosahedral clusters including [NiPb_12_]^2–^ and [CoPb_12_]^3–^.^13,14^ The same [CuPb_12_]^−^ ion was also observed observed in the ESI-MS of [Cu_4_Pb_18_]^4–^, in this case alongside the parent ion, [Cu_4_Pb_18_]^−^.^44^ The fact that [Cu_4_Pb_22_]^4–^ and [Cu_4_Pb_18_]^4–^ have similar ESI-MS fingerprints hints at a dynamic situation in solution, where interconversion between clusters with different Cu/Pb ratios is facile.

### Pathway for Cluster Growth

**Figure 4:**
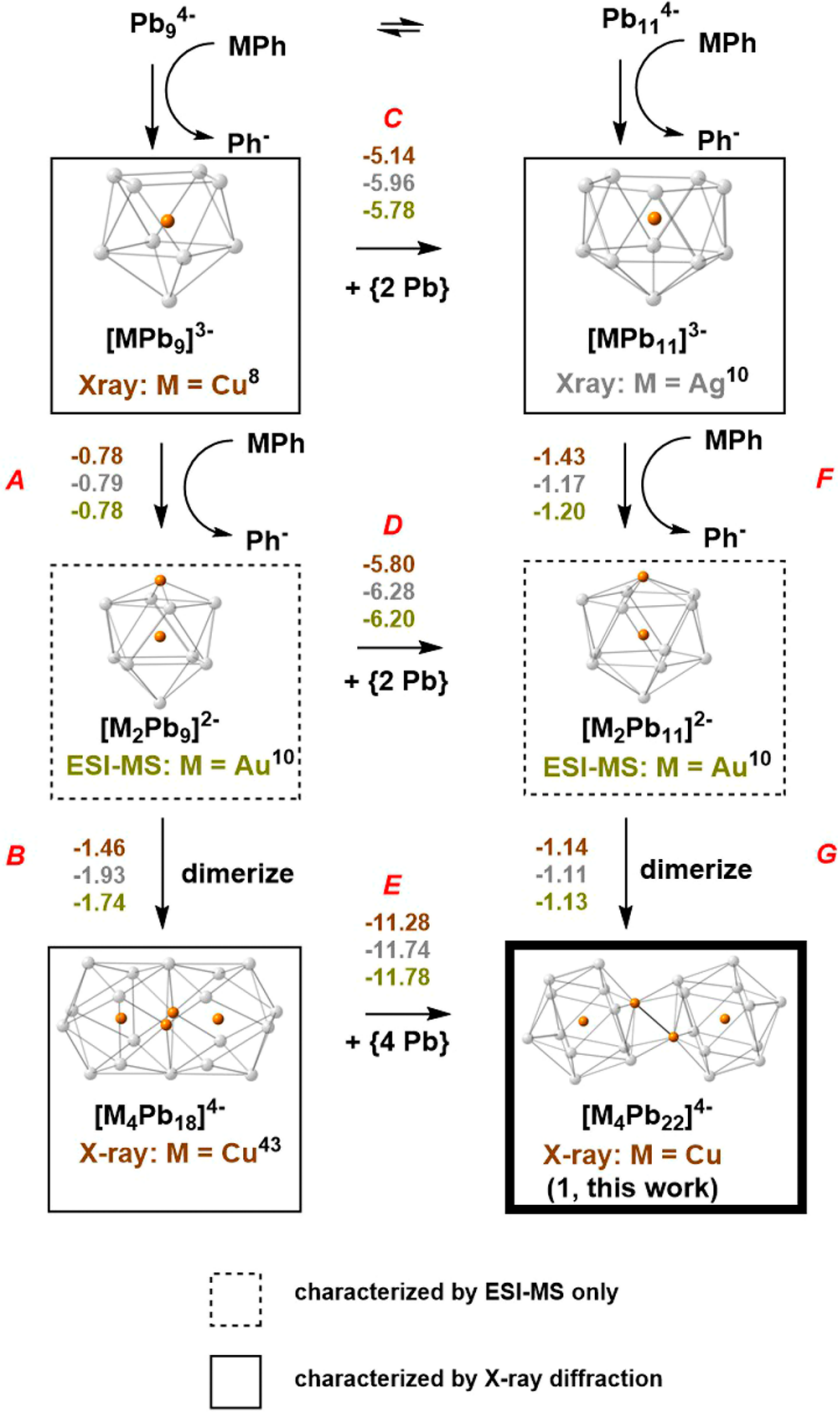
Proposed fragment assembly pathway for [Cu_4_Pb_22_]^4–^ and [Cu_4_Pb_18_]^4–^ Clusters that have been crystallographically characterized are boxed, those that have been identified by ESI-MS (composition only) are in dashed boxes. The new compound reported in this paper, [Cu_4_Pb_22_]^4–^, is picked out in a bold box. The triad of numbers (colored copper, silver, and gold for Cu, Ag, and Au) above/beside each step labeled A–G represent the calculated reaction energies (in eV).

The characterization of the new cluster reported in this paper, K([2.2.2-crypt)]_4_[Cu_4_Pb_22_], (1), expands the already extensive body of data on coinage-metal clusters of Pb that has been reported in previous papers, both by us^11,12^ and by other authors.^9,16^ Our aim here is to collate all of the available data, both new and previously published, into a coherent model for cluster growth. A possible pathway for the growth of Pb clusters of the coinage metals, leading to [Cu_4_Pb_22_]^4–^, is presented in Figure 4, where the group-11 atom is represented generically as “M”. The first column (steps A and B) terminates at [M_4_Pb_18_]^4–^, a known compound for M = Cu,^44^ while the second column (F/G) terminates at [M_4_Pb_22_]^4–^, the new Cu species reported for the first time here. Our working hypothesis is that if a cluster has either been characterized by X-ray crystallography or observed in the ESI-MS for one member of the Cu/Ag/Au triad, it is not unreasonable to propose its existence, even if only as a transient intermediate, for the others. In cases where clusters have been structurally characterized with one specific member of group 11, the cluster is enclosed in a box and the metal in question is identified, for example, by “X-ray: M = Cu” in the case of [MPb_9_]^3–^, where the only available experimental evidence comes from ESI-MS; the cluster is enclosed in a dashed box and the particular metal is identified as “ESI-MS: M = Au”, as for example in the case of [M_2_Pb_9_]^2–^. Balanced equations for each of the steps in the Figure are given in Table 1.

**Table 1:** Balanced Equations for Steps A–G in Figure 4

A	[MPb_9_]^3–^	+	MPh	→	[M_2_Pb_9_]^2–^	+	Ph–
B	2[M_2_Pb_9_]^2–^			→	[M_4_Pb_18_]^4–^		
C	[MPb_9_]^3–^	+	2Pb	→	[MPb_11_]^3–^		
D	[M_2_Pb_9_]^2–^	+	2Pb	→	[M_2_Pb_11_]^2–^		
E	[M_4_Pb_18_]^4–^	+	4Pb	→	[M_4_Pb_22_]^4–^		
F	[MPb_11_]^3–^	+	MPh	→	[M_2_Pb_11_]^2–^	+	Ph–
G	2[M_2_MPb_11_]^2–^			→	[M_4_Pb_22_]^4–^		

### Analysis of the Fundamental Steps Using DFT

Before exploring the energetics of the various steps in Figure 4, it is important to highlight the approximations and assumptions that underpin our computational model. In the synthetic chemistry described here and elsewhere,^11^ the source of the coinage metal is a mesityl compound, either in the form of a cluster ((AgMes)_4_^11^) or as a phosphine complex (AuMes(PPh_3_)^11^ or, here, CuMes(PPh_3_)_2_). To balance the equations in Table 1, we use the simplified model fragment MPh (Ph = phenyl) as the source of metal (the methyl groups of mesityl are removed for computational expedience) and we assume that the Ph^–^ anion is released into solution as the metal is incorporated into the cluster. In fact it is likely that the mesityl anion abstracts a proton from the ethylenediamine solvent in the course of these reactions,^47^ but the precise fate of the ligand is not critical to the arguments we make here because whatever approximations are made in modeling the ligand are, they are the same for all three coinage metals. For this reason, our emphasis throughout this discussion is on the *relative* energetics of the Cu/Ag/Au triad, rather than on any one specific reaction. The two columns in Figure 4 are connected by cluster expansion reactions, C, D and E that increase the Pb/M ratio while retaining the same number of coinage metal ions. As was the case for the fate of the mesityl anion, it is difficult to establish the source of additional Pb atoms in order to balance the chemical reactions: they may, for example, be extruded from the Pb_9_^4–^ Zintl ions, or from fragments of these larger clusters, or indeed from nanoparticles of elemental Pb, which have been observed in reactions of this kind.^33^ Again, this is not a significant limitation as long as our emphasis remains on the *relative* energetics within the Cu/Ag/Au series, where any deficiencies in the treatment of the Pb atoms in our computational model are at least constant. In the following analysis, we choose the energy of a free Pb atom in its triplet ground-state (6s^2^6p^2^) as a convenient reference. For each step in Figure 4, three energies are given corresponding to the balanced equations for the reaction with M = Cu, Ag, and Au (the colors in the Figure correspond to the elements). Absolute energies of all reported species are collected in the Supporting Information, Tables S3 and S4.

### Formation of the Icosahedral Fragments, [M_2_Pb_11_]^2–^

**Figure 5:**
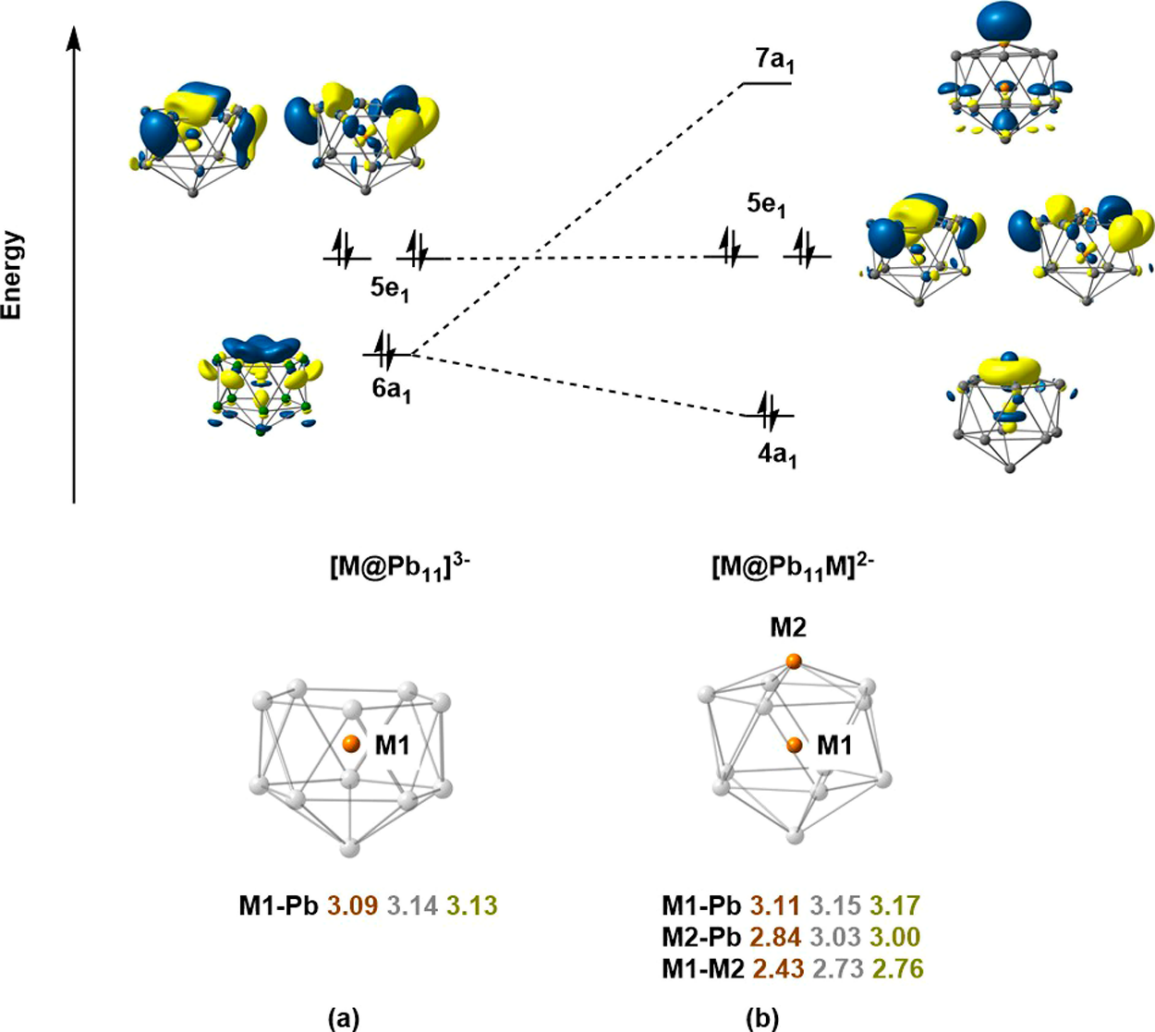
Canonical Kohn–Sham orbitals illustrating the binding of M^+^ to the pentagonal face of [MPb_11_]^3–^. Averaged bond lengths (in Å) for M = Cu, Ag, and Au are shown below: values are color-coded according to the color of the element.

The initial stages of cluster growth involve the reaction of K_4_Pb_9_ with the organometallic source of low-valent copper, leading to either the known compound [CuPb_9_]^3–^ (reaction A) or the larger [CuPb_11_]^3–^ (reaction F), the copper analogue of known nido-[AgPb_11_]^3–^. While [CuPb_11_]^3–^ has not been isolated, Eichhorn’s recent synthesis of [(Cp*Ru)CuPb_11_]^2–^ offers independent support for its existence in solution.^16^ We do not address here the question of how these initial endohedral fragments form, although it is likely that smaller transient components such as tetrahedral Pb_4_^4–^ play a role, as proposed by Dehnen and Weigend in their study of Ta/Ge/As clusters.^17^ The next step involves the trapping of a second Cu^+^ ion to form either bicapped square antiprismatic [Cu_2_Pb_9_]^2–^ (B) or icosahedral [Cu_2_Pb_11_]^2–^ (G). Although the only direct evidence for the existence of icosahedral clusters comes from ESI-MS, the presence of the M_2_Pb_11_ unit in [Au_8_Pb_33_]^6–^ and [Au_12_Pb_44_]^8–^^11^ and now also in [Cu_4_Pb_22_]^4–^ suggests that it is an important intermediate. The optimized structure of the [Cu_2_Pb_11_]^2–^ anion is shown in Figure 5, and the total binding energy (Δ*E*_total_) for the Cu^+^ cation at the [CuPb_11_]^3–^ fragment is shown in the first column of Table 2, along with its component parts according to the energy decomposition scheme proposed by Ziegler and Rauk.^43^ Note that the total binding energies for M^+^ differ from the energies for step F in Figure 4, where the reaction in question is [MPb_11_]^3–^ + MPh → [M_2_Pb_11_]^2–^ + Ph^–^ rather than [MPb_11_]^3–^ + M^+^ → [M_2_Pb_11_]^2–^. In Table 2, Δ*E*_prep_ is the difference in energies between the two fragments in their optimized geometries and the geometries they adopt in the cluster, Δ*E*_steric_ is the sum of Pauli and electrostatic energies, Δ*E*_orbital_ is the energy from interaction of occupied and virtual orbitals on the two fragments (decomposed into separate contributions from the irreducible representations of the *C*_5*v*_ point group), and ΔΔ*E*_solvation_ is the difference between solvation energies of the cluster and its component fragments.

**Table 2:** Decomposition of Binding Energies (in eV) for Metal Cations, M′^+^, with [MPb_11_]^3–^a^,^^43^

	M/M′+			
	Cu/Cu^+^	Cu/[CpRu]^+^	Ag/Ag^+^	Au/Au^+^
Δ*E*_prep_	+0.19	+0.48	+0.19	+0.38
Δ*E*_steric_	–11.30	–7.80	–10.83	–11.07
Δ*E*_orbital_	–5.14	–7.73	–4.09	–6.28
***a*_1_**	**–2.67**	**–1.18**	**–2.25**	**–3.90**
*a*_2_	0.00	0.00	0.00	0.00
***e*_1_**	**–1.78**	**–5.68**	**–1.38**	**–1.65**
*e*_2_	–0.69	–0.87	–0.46	–0.73
ΔΔ*E*_solvation_	+10.74	+9.78	+10.48	+10.43
Δ*E*_total_	–5.51	–5.27	–4.25	–6.54

a Important terms are picked out in bold font.

The energy decomposition analysis confirms that the orbital interaction between the two fragments, [CuPb_11_]^3–^ and Cu^+^, is dominated by the *a*_1_ representation, and specifically the interaction of the 6*a*_1_ orbital of the *nido* cluster with the empty 4s orbital of Cu^+^, the latter making the dominant contribution to the 7*a*_1_ LUMO of [Cu_2_Pb_11_]^2–^. The absence of a pair of electrons in this orbital leaves the total valence electron count at 48, 2 fewer than the 4*n* + 2 = 50 expected for a stable *closo* icosahedron and renders the cluster substantially Lewis acidic. However, the doubly degenerate 5*e*_1_ HOMO of the *nido* cluster, [CuPb_11_]^3–^, does not participate in the binding of Cu^+^ to the open face because the coinage metal cation lacks low-lying vacant orbitals of appropriate symmetry (the lowest of *e*_1_ symmetry are the 4p). As a result, the HOMO of [Cu_2_Pb_11_]^2–^ is non-bonding with respect to the capping atom, and its high energy confers significant Lewis basic character on the cluster. In short, the [Cu_2_Pb_11_]^2–^ unit has amphoteric character: it is simultaneously Lewis acidic and Lewis basic, and this proves critical both to the subsequent dimerization step (*G*) and to the nucleation of additional metal atoms.

It is instructive at this point also to compare the binding of the [CuPb_11_]^3–^ fragment to a Cu^+^ ion with the corresponding process with the [RuCp*]^+^ fragment found in the stable cluster [(Cp*Ru)CuPb_11_]^2–^.^16^ The frontier orbital array of [CuPb_11_]^3–^ shown in Figure 5a establishes an isolobal relationship between it and the cyclopentadienyl anion, Cp^–^, and indeed [(Cp*Ru)CuPb_11_]^2–^ can be understood as being isolobal with ruthenocene, Cp*_2_Ru. This isolobality is based on the presence of three high-lying orbitals, 6*a*_1_ and 5*e*_1_, and, critically, all three participate in bonding the cluster to the [RuCp*]^+^ fragment which, unlike a Cu^+^ ion, does have low-lying vacant orbitals of *e*_1_ symmetry (Supporting Information, Figure S5). The total binding energies for the Cu^+^ and [RuCp*]^+^ fragment shown in Table 2 are quite similar (−5.51 and −5.27 eV, respectively), but the decomposition of the orbital component reveals a dominant contribution from the *e*_1_ representation (−5.68 eV) in the latter. The [RuCp*]^+^ fragment therefore stabilizes the 5*e*_1_ orbital on the [CuPb_11_]^3–^ unit in a way that Cu^+^, or indeed any other metal cation with a d^10^ configuration, does not.

### Dimerization to [Cu_4_Pb_22_]^4–^

**Figure 6:**
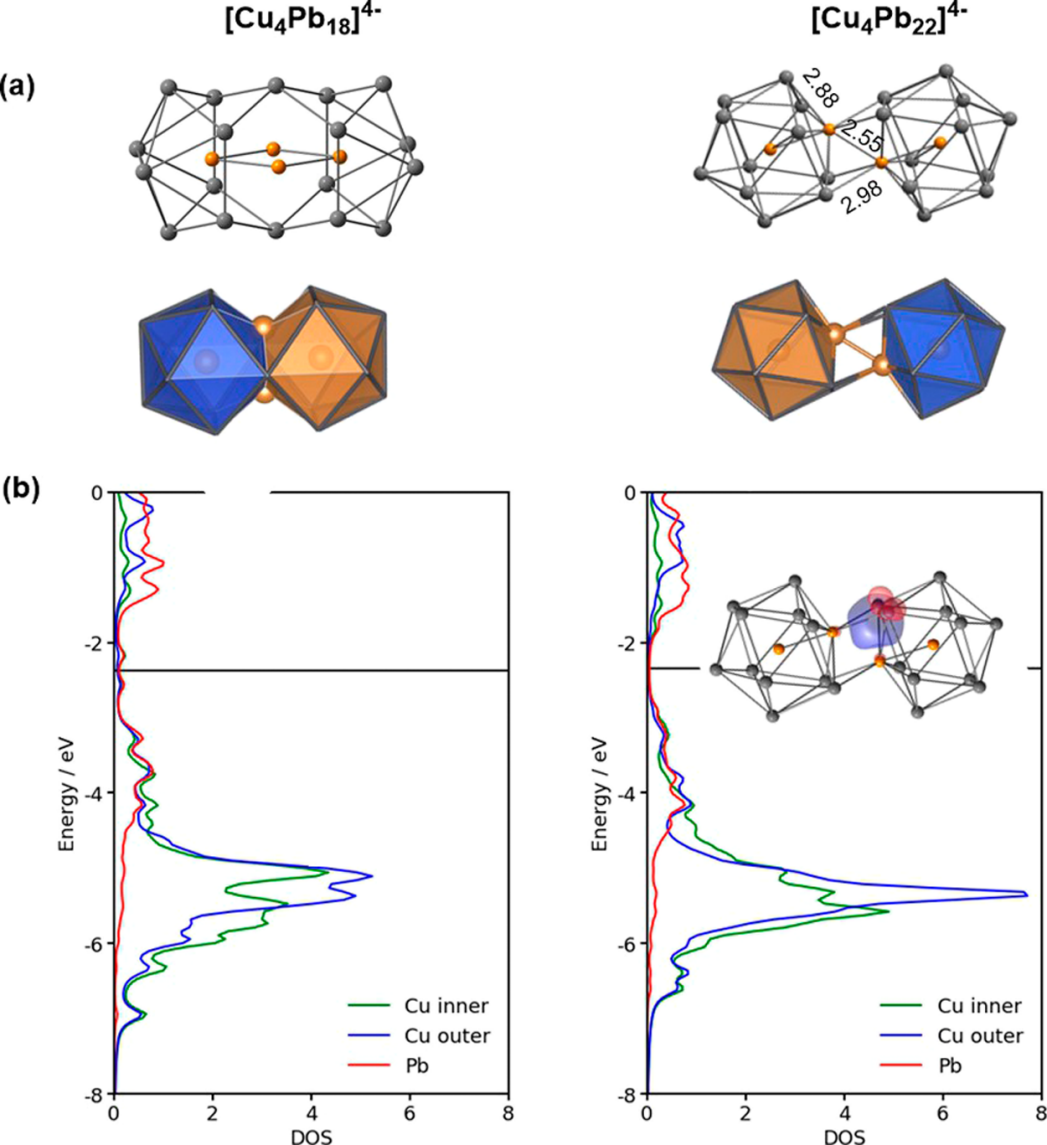
Perspectives on the electronic structure of [Cu_4_Pb_22_]^4–^ and [Cu_4_Pb_18_]^4–^. (a) Ball-and-stick representation (above) and polyhedral representations of the structure, with optimized parameters for [Cu_4_Pb_22_]^4–^. (b) Projected density of states. A localized orbital is shown inset for [Cu_4_Pb_22_]^4–^ only.

The optimized geometry of the title cluster, [Cu_4_Pb_22_]^4–^, is shown in Figure 6 and is fully consistent with the X-ray data summarized in Figure 2. The frontier canonical orbital domain of [Cu_2_Pb_11_]^2–^ shown in Figure 5b, with a doubly degenerate HOMO and a low-lying vacant LUMO of *a*_1_ symmetry, suggests an isolobal relationship to BH_3_ which, in turn, highlights the isolobal relationship between the title cluster, [Cu_4_Pb_22_]^4–^, and diborane, B_2_H_6_ (Supporting Information, Figure S7). Donor–acceptor interactions from one component of the 5*e*_1_ HOMO of one fragment to the 7*a*_1_ LUMO of the other stabilize the Cu_2_Pb_4_ trigonal antiprism linking the two subunits. The dimerization results in a substantial re-hybridization that complicates a simple fragment-based analysis of the canonical orbitals, but orbital localization (using the Pipek-Mezey algorithm) confirms the presence of two 3-center-2-electron bonds linking the two sub-units (Figure 6b, inset). These localized orbitals resemble closely those that bind the Cu_2_Sn_4_ antiprism in [Cu_2_Sn_10_Sb_6_]^4–^,^46^ and indeed the total dimerization energy of −1.14 eV is very close to the value of −1.01 eV reported in that case. The very short Cu–Pb bond lengths within the trigonal antiprism (2.98 Å from DFT, Figure 6, ∼2.90 Å in the X-ray structure, Figure 2) are testament to the strength of the 3-center-2-electron bonds, as is the elongation of the Cu–Cu distances in the icosahedral units (2.54 Å in the dimer *vs* 2.42 Å in the isolated fragments). Ultimately, the tendency to form a dimer can be traced to the fact that the electron donor capacity of the doubly degenerate 5*e*_1_ HOMO is not saturated by the binding of a Cu^+^ ion to the open Pb_5_ face, as a result of which the icosahedral [Cu_2_Pb_11_]^2–^ unit remains “sticky”. It is no coincidence, then, that Cu^+^ is the common denominator in the other two known “dimers of deltahedra”, [Cu_2_Sn_10_Sb_6_]^4–^ and [Cu_2_Ge_18_Mes_2_]^4–^.

### Factors Influencing Cluster Expansion: [Cu_4_Pb_22_]^4–^*Versus* [Cu_4_Pb_18_]^4–^

We noted in the introduction the potential importance of synthetic routes that lead to clusters with a precisely defined ratio of transition and main-group metals. The isolation of both [Cu_4_Pb_18_]^4–^ and [Cu_4_Pb_22_]^4–^ therefore poses an important question: what factors control the formation of one over the other? Our discussion of the fundamental steps in Figure 4 has focused on the pathway linking [CuPb_11_]^3–^ to the title cluster, [Cu_4_Pb_22_]^4–^ (F–G), but we can identify very similar patterns in the early stages of the pathway linking [CuPb_9_]^3–^ to [Cu_4_Pb_18_]^4–^ (steps A–B). The electronic structure of the bicapped square antiprismatic [CuPb_9_Cu]^2–^ unit (Supporting Information, Figure S6) is strikingly similar to that of [Cu_2_Pb_11_]^2–^: the LUMO has dominant 4s character on the Cu^+^ ion on the cluster surface, while the doubly degenerate HOMO is localized on the adjacent square face. The cluster is therefore also amphoteric and a dimerization step analogous to that discussed above for [Cu_4_Pb_22_]^4–^ would generate a *C*_2*h*_-symmetric structure where the two bicapped square antiprisms are linked *via* a Cu_2_E_4_ trigonal antiprism, precisely the motif found in the [Cu_2_Ge_18_Mes_2_]^4–^ (Figure S8).^45,46^ Instead, however, the dimerization goes a step further, to the point where the two Cu_2_Pb_9_ units fuse to form a single continuous *D*_2*h*_-symmetric Pb_18_ cage, also shown in Figure 6. Both isomers are local minima on the potential energy surface, but the experimentally observed *D*_2*h*_-symmetric structure is the more stable of the two by 0.76 eV, indicating that the driving force to coalesce to a single Pb_18_ cage is substantial. Despite this, the projected density of states (PDOS) plots shown in Figure 6b reveal no significant differences in electronic structure between the two clusters: both feature well-separated maxima for the Cu 3d and Pb 6s/6p manifolds with no evidence for substantial Cu 3d-Pb covalency. The two clusters do, however, share a common icosahedral coordination geometry about the encapsulated Cu^+^ ion: Cu@CuPb_11_ in [Cu_4_Pb_22_]^4–^ and Cu@Cu_2_Pb_10_ in [Cu_4_Pb_18_]^4–^. It is possible, then, that the inherent stability of the icosahedron is the controlling feature, and that coalescence of two endohedral MPb_*x*_ fragments, whatever their size, will proceed to the point where an icosahedral geometry is achieved. In such circumstances, it seems likely that the product distribution can only be controlled by varying the Cu/Pb ratio in solution.

### Periodic Trends in Cluster Growth: Comparison of Cu, Ag, and Au

The cluster growth pathway proposed in Figure 4 and the orbital analysis in Figure 5 are applicable to the coinage metals in general, but there are nevertheless some conspicuous differences between Cu, Ag, and Au that merit comment. The first of these is that the smallest *nido*-[MPb_11_]^3–^ unit has been isolated only for Ag: for both Cu and Au, further metal cations bind to the open Pb_5_ face. The trend in total binding energies of a metal cation to [M_2_Pb_11_]^2–^ ([MPb_11_]^3–^ + M^+^ → [M_2_Pb_11_]^2–^), shown in the final row of Table 2, correlates with the gas-phase ionization energies of the metals (7.73, 7.58, and 9.23 eV for Cu, Ag, and Au, respectively^48^) with the values for Ag being conspicuously lower than those for either Cu or Au. The relatively high energy of the 5s orbital of Ag, and the consequent weak binding of the Ag^+^ cation to the Pb_5_ face, offers an immediate explanation for the isolation of *nido*-[AgPb_11_]^3–^ but not its Cu or Au analogues. The fact that *nido*-[AgPb_11_]^3–^ can be isolated presents the intriguing possibility that ternary clusters of the form [AgM′Pb_11_]^2–^ might be accessible through further reaction of [AgPb_11_]^3–^ with a source of M′^+^. The total cation binding energies for all possible combinations of M and M′ are summarized in Table 3 (note that the diagonal elements of this Table are the total energies given in Table 2). For a given M′^+^, the binding becomes stronger in the order Au^+^ ∼ Ag^+^ < Cu^+^, reflecting a slight expansion of the open face of *nido*-[MPb_11_]^3–^ as M gets larger, but the ability to bind M′^+^ to the open face is, to a good approximation, independent of the identity of the endohedral metal. The strongest binding of the capping ion is therefore found for [CuPb_11_Au]^2–^, but Au^+^ binds strongly in all cases and ternary clusters based on [AgPb_11_Au]^2–^ icosahedra appear to be realistic synthetic targets. Our attempts to synthesize clusters of this type have, thus far, been frustrated by the relatively small amounts of [AgPb_11_]^3–^ available as the starting material.

**Table 3:** Total Binding Energies, Δ*E*_total_, (in eV) for Coinage Metal Cations, M′^+^, with [MPb_11_]^3–^a

		M^′^+		
		Cu	Ag	Au
M	Cu	–5.51	–4.44	–6.88
	Ag	–5.22	–4.24	–6.60
	Au	–5.25	–4.25	–6.57

a M denotes the endohedrally encapsulated metal; M′ denotes the metal on the surface.

The energies of the cluster expansion reactions (steps C, D, and E in Figure 4) show a rather different pattern: the reactions becoming more favorable in the order Cu < Ag ∼ Au, correlating approximately with the size of the transition metal ion (*r*{Cu^+^} = 0.97 Å, *r*{Ag^+^} = 1.29 Å, *r*{Au^+^} = 1.33 Å according to Shannon’s revised tables^49^), rather than with the ionization energies. This is intuitive: expansion of the Pb_*x*_ cluster in steps C, D, and E becomes increasingly favorable as the radius of the endohedral cation becomes larger, and the clusters [MPb_9_]^3–^ and [M_4_Pb_18_]^4–^ are for this reason likely inaccessible for all but the smallest of the coinage metals, Cu.

The most conspicuous difference between the chemistries of Cu, Ag, and Au in these reactions is, however, the fact that we isolate larger clusters which incorporate neutral metal atoms, M, *only* in the case of Au.^11^ The absence of equivalent clusters of Ag in the present case can probably be traced to the weak binding of Ag^+^ to the open pentagonal face of [AgPb_11_]^3–^ discussed above, which immediately rules out further condensations based on icosahedral subunits. In the subsequent analysis, we therefore focus on the comparison between Cu and Au, both of which have been shown to form clusters based on the icosahedral building block, M_2_Pb_11_. One possible explanation for the absence of larger clusters for Cu is that the new dimer reported here, [Cu_4_Pb_22_]^4–^, is a thermodynamic sink that prevents further reactions. However, the energies of step G in Figure 4 are almost independent of the identity of the metal, suggesting that the dimerization step does not effectively differentiate members of the triad. Alternatively, the answer may lie in the more facile reduction of Au^+^ to Au and its stronger binding to the icosahedral subunits, a topic that we explore in the following paragraphs.

**Figure 7:**
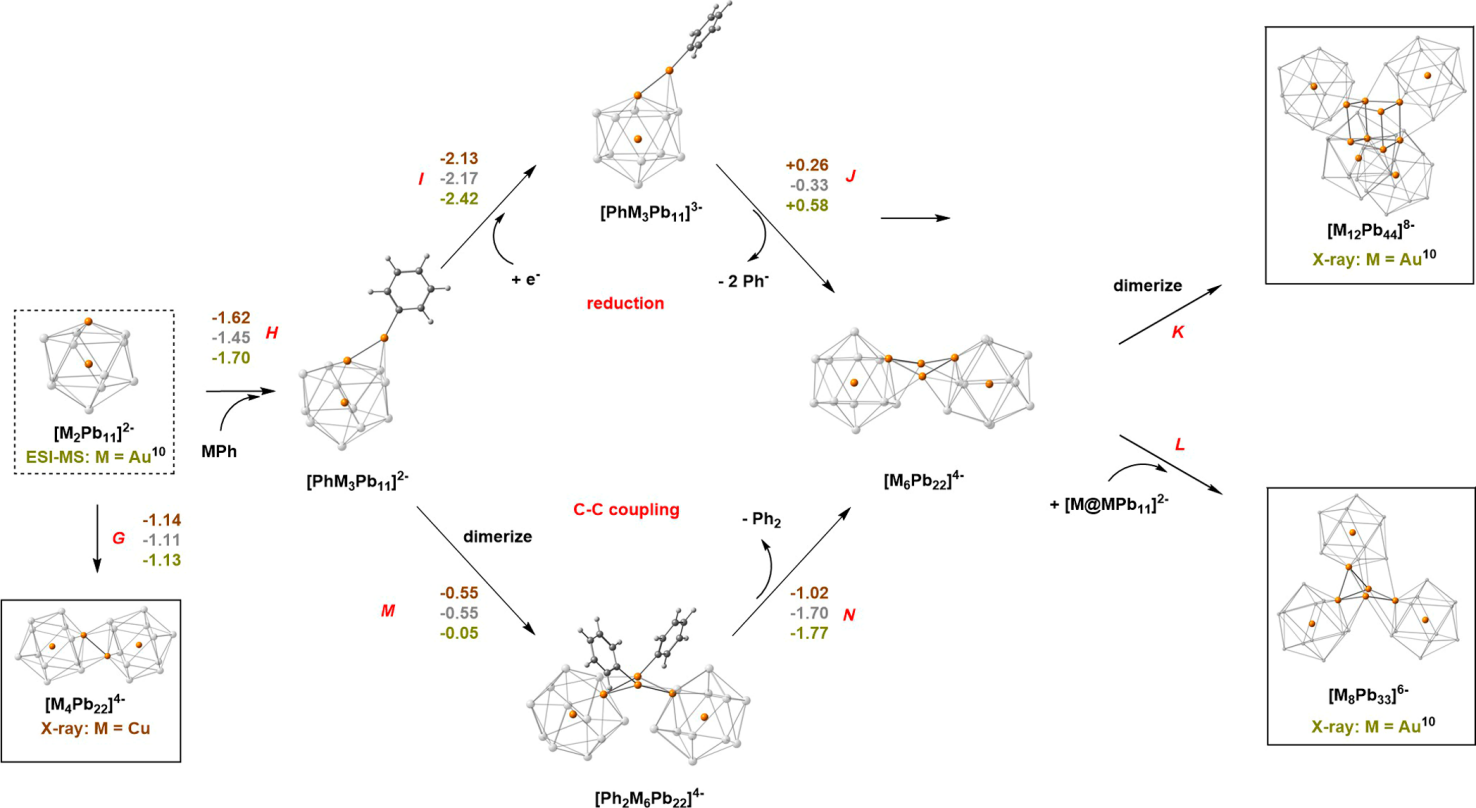
Proposed fragment assembly pathway leading to metal clusters containing neutral M. Clusters that have been crystallographically characterized are boxed, those that have been identified by ESI-MS (composition only) are in dashed boxes. The triad of numbers (colored copper, silver, and gold for Cu, Ag, and Au) above/beside each step represent the calculated reaction energies (in eV).

The amphoteric nature of the 48-electron [M_2_Pb_11_]^2–^ icosahedra that led to dimerization in the case of [Cu_4_Pb_22_]^4–^ can also lead to the binding of MMes fragments to the cluster surface, at which point reduction (possibly by residual Pb_9_^4–^,^50^) followed by loss of Mes^–^ appears a plausible route to formation of [Au_6_Pb_22_]^4–^ and hence to [Au_12_Pb_44_]^8–^ and [Au_6_Pb_33_]^6–^. This possibility is explored in the upper part of Figure 7 (labeled “reduction”), where again the mesityl ligands are modeled by the more tractable phenyl unit. Balanced equations for the key reaction steps are shown in Table 4. In the first step (H), the MPh fragment binds to the MPb_5_ face of the icosahedron with, in the case of M = Au, short Au–Au and Au–Pb distances of 2.80 and 3.06 Å, respectively. This is then followed by a one-electron reduction (step I), that is most exothermic for Au, and then loss of a Ph^–^ ligand and dimerization to form [M_6_Pb_22_]^4–^ (step J). A significant point is that the corresponding energies for one-electron reduction of the clusters in the absence of the absorbed MPh fragment (*i.e.,* [M_2_Pb_11_]^2–^ → [M_2_Pb_11_]^3–^) are less exothermic by *ca* 0.4 eV (Δ*E* = −1.72 and −2.10 eV for Cu and Au, respectively): the binding of the MPh unit to the surface clearly renders the cluster more susceptible to reduction.^50^ An alternative possibility, that does not involve an external reducing agent, is that the mesityl ligands may be lost *via* C–C coupling reactions, forming bimesityl,^51,52^ for which there is precedent in the literature.^53−55^ This “C–C coupling” pathway is explored in the lower half of Figure 7, and leads to the same intermediate M_6_ cluster, [M_6_Pb_22_]^4–^, *via* the sequence (H → M → N). The reaction again involves the binding of MPh, now followed by dimerization (step M) and reductive elimination of Ph_2_ (step N). It is striking that in both pathways that the step that differentiates Cu from Au most clearly is the one where the metal is reduced, either by an external reducing agent (step I) or *via* electron transfer from the bound phenyl ligands (step N). Indeed the difference between Cu and Au is approximately twice as large in the latter, where two Au^+^ are reduced. Without additional experimental evidence, it is not easy to distinguish between these two pathways, but the computed activation energy for the C–C coupling reaction (step N) is 1.18 eV (50.1 kcal/mol, see Supporting Information, Figure S9), somewhat higher than the values of 31–39 kcal/mol reported by Boronat *et al.*(53,54) and certainly rather high for reactions that occur in the range of 40–60 °C.^11^ On this basis, reduction by the starting material, Pb_9_^4–^, with concomitant loss of Mes^–^, seems the more plausible route. Whichever mechanism dominates, it is clear that the process is more favorable for Au than it is for Cu due to the greater ease of reduction that is manifested in the greater exothermicity of steps I or N in Figure 7. The precise balance between dimerization to form [M_4_Pb_22_]^4–^ (step G) and the formation of larger clusters will depend critically on the concentrations of the various species in solution, but the computed energy landscape suggests that nucleation of metal atoms on the surface of the cluster is more likely to prevail for Au than for Cu.

**Table 4:** Balanced Equations for Steps H–N in Figure 7

H	[M_2_Pb_11_]^2–^ + MPh	→	[PhM_3_Pb_11_]^2–^
I	[PhM_3_Pb_11_]^2–^ + *e*^–^	→	[PhM_3_Pb_11_]^3–^
J	2[PhM_3_Pb_11_]^3–^	→	[M_6_Pb_22_]^4–^ + 2Ph^–^
K	2[M_6_Pb_22_]^4–^	→	[M_12_Pb_44_]^8–^
L	[M_6_Pb_22_]^4–^ + [M_2_Pb_11_]^2–^	→	[M_8_Pb_33_]^6–^
M	2[PhM_3_Pb_11_]^2–^	→	[Ph_2_M_6_Pb_22_]^4–^
N	[Ph_2_M_6_Pb_22_]^4–^	→	[M_6_Pb_22_]^4–^ + Ph_2_

## Summary and Conclusions

In this paper, we have reported the isolation and structural characterization of the [Cu_4_Pb_22_]^4–^ cluster, the gold analogue of which was previously postulated to be the “missing link” in the growth of larger Au_*x*_Pb_*y*_ clusters such as [Au_8_Pb_33_]^6–^ and [Au_12_Pb_44_]^8–^.^11^ The cluster itself is a dimer of [Cu_2_Pb_11_]^2–^ icosahedra linked *via* a Cu_2_Pb_4_ trigonal antiprism, the stability of which stems from strong donor–acceptor interactions between the Pb-centered HOMO of one icosahedral unit and the Cu 4s-based LUMO of the other. The system is, in fact, isolobal with B_2_H_6_, and the bonding shares much in common with this simple molecule.

The tendency of the [M_2_Pb_11_]^2–^ icosahedra to dimerize or even oligomerize appears to be a general feature of the coinage metals (M = Cu, Au), which stand apart from the apparently closely-related MPb_12_ systems, none of which behave in the same way. The unique ability of the coinage metal clusters to dimerize and oligomerize is a direct consequence of their *n*d^10^(*n* + 1)s^0^ configuration: the vacant (*n* + 1)s orbital confers a high degree of Lewis acidity, while the absence of vacant *n*d orbitals leaves the Pb-based HOMO high in energy and available to act as a Lewis base. This amphoteric character also allows the clusters to act as a nucleation site for additional zerovalent metal atoms, which leads to the agglomeration of larger clusters [Au_8_Pb_33_]^6–^ and [Au_12_Pb_44_]^8–^. The 5s orbital of Ag is higher in energy than either the 4s of Cu or the 6s of gold, and as a result, the chemistry of Ag stands out as quite distinct in that the only isolated product is *nido*-[AgPb_11_]^3–^, where a second Ag^+^ cation does not bind at the open face. The contrasting Zintl cluster chemistry of the Cu/Ag/Au triad is, therefore, an elegant illustration of the alternation of periodic properties commonly encountered in this region of the periodic table.

## Supporting Information Available

Black details of crystal data and structural refinement, asymmetric unit of the crystal and views of the unit cell, EDX spectrum and analysis. The computational analysis is expanded to include Kohn-Sham MO diagrams, optimized structures of isomers, a discussion of isolobal relationship, the transition state for C-C coupling, formation energies of metal atoms and cations, and a summary of total formation energies of all clusters and fragments (PDF)

## Accession Codes

CCDC 2054778 contains the supplementary crystallographic data for this paper. These data can be obtained free of charge via www.ccdc.cam.ac.uk/data_request/cif, or by emailing data_request@ccdc.cam.ac.uk, or by contacting The Cambridge Crystallographic Data Centre, 12 Union Road, Cambridge CB2 1EZ, UK; fax: +44 1223 336033.
