# Supplementary material for: Missing Link in the Growth of Lead-Based Zintl Clusters: Isolation of the Dimeric Plumbaspherene [Cu4Pb22]4–
Source: J Am Chem Soc. 2022 Apr 22;144(18):8007–17. doi: 10.1021/jacs.1c10106 (PMC9100666; doi:10.1021/jacs.1c10106)
Supplement: Supplementary file 1 — ja1c10106_si_001.pdf [file ja1c10106_si_001.pdf]

Supporting Information

A missing link in the growth of lead-based Zintl clusters: isolation of the dimeric plumbaspherene $[Cu_4Pb_{22}]^{4-}$.

Harry W. T. Morgan and John E. McGrady, Department of Chemistry, University of Oxford, South Parks Road, Oxford OX1 3QR, U.K.

Cong-Cong Shu and Zhong-Ming Sun, State Key Laboratory of Elemento-Organic Chemistry, Tianjin Key Lab of Rare Earth Materials and Applications, School of Material Science and Engineering, Nankai University, Tianjin 300350, China.

Supplementary crystallographic information

Figure S1 shows a photograph of the black block-like crystals of K(2,2,2−crypt)$_4$[Cu$_4$Pb$_{22}$] while Table S1 contains a summary of the diffraction data data.

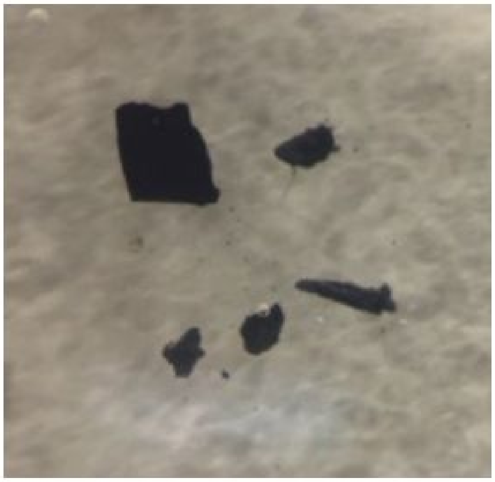

Figure S1: Black block crystals of [K(2,2,2−crypt)]$_4$[Cu$_4$Pb$_{22}$].

Table S1: Details of Crystal Data and Structural Refinement.

Identification code	K(2,2,2−crypt)$_4$[Cu_4Pb_{22}]
Empirical formula	$C_{36}H_{72}N_4O_{12}K_2Cu_2Pb_{11}$
Formula weight	3237.35
Temperature/K	100.00
Crystal system	monoclinic
Space group	$C2/c$
a/Å	29.7576(3)
b/Å	16.12050(10)
c/Å	27.6598(2)
α/°	90.00
β/°	105.2760(10)
γ/°	90.00
Volume/Å^3	12799.80(18)
Z	8
ρ_{calc}g/cm^3	3.360
μ/mm^{-1}	57.199
F(000)	11280.0
2θ range for data collection/°	7.48 to 134
Reflections collected	28630
Data/restraints/parameters	11387/1614/604
Goodness-of-fit on F_2	1.085
Final R indices [I≥2σ (I)]	$R_1 = 0.0669, wR_2 = 0.1865$
Final R indices [all data]	$R_1 = 0.0700$, $wR_2 = 0.1900$
Largest diff. peak/hole / e Å^{-3}	6.46/-3.63

R$_1$=$\sum ||F_0| - |F_c||/\sum |F_0|$, wR$_2$=$(\sum w[(F_0)^2 - (F_c)]^2/\sum w[(F_0)^2]^2)^{1/2}$
GooF=$(\sum w[(F_0)^2 - (F_c)]^2/$(n-p)$)^{1/2}$

Figures S2 and S3 show the asymmetric unit of the crystal and views of the unit cell down the a, b and c axes, respectively.

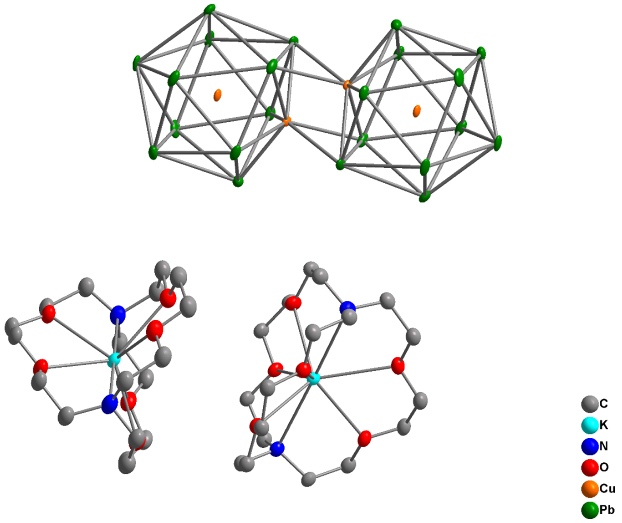

Figure S2: Asymmetric unit of $[K(2,2,2-crypt)]_4[Cu_4Pb_{22}]$ with the cluster fragment. Thermal ellipsoids are drawn at 50% probability. The minor components are omitted for clarity.

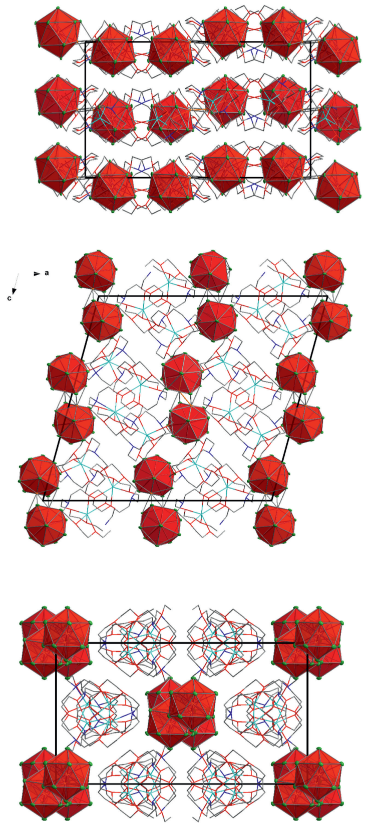

Figure S3: Unit cell of $[K(2,2,2{-}crypt)]_4[Cu_4Pb_{22}]$ viewed along the a, b and c axes. Minor components in the cluster site are omitted for clarity.

Energy dispersive X-ray (EDX) spectroscopy

The spectrum and its analysis are collected in Figure S4 and Table S2. The analysis indicates that the atom% of Cu and Pb are within 5% of the ideal values. Deviations of this magnitude from compositions calculated based on the formulation confirmed by single crystal X-ray diffraction are often observed in Zintl-ion chemistry. It is possible that the marginal excess of Cu reflects partial decomposition of the surface of the crystal after lengthy exposure to air.

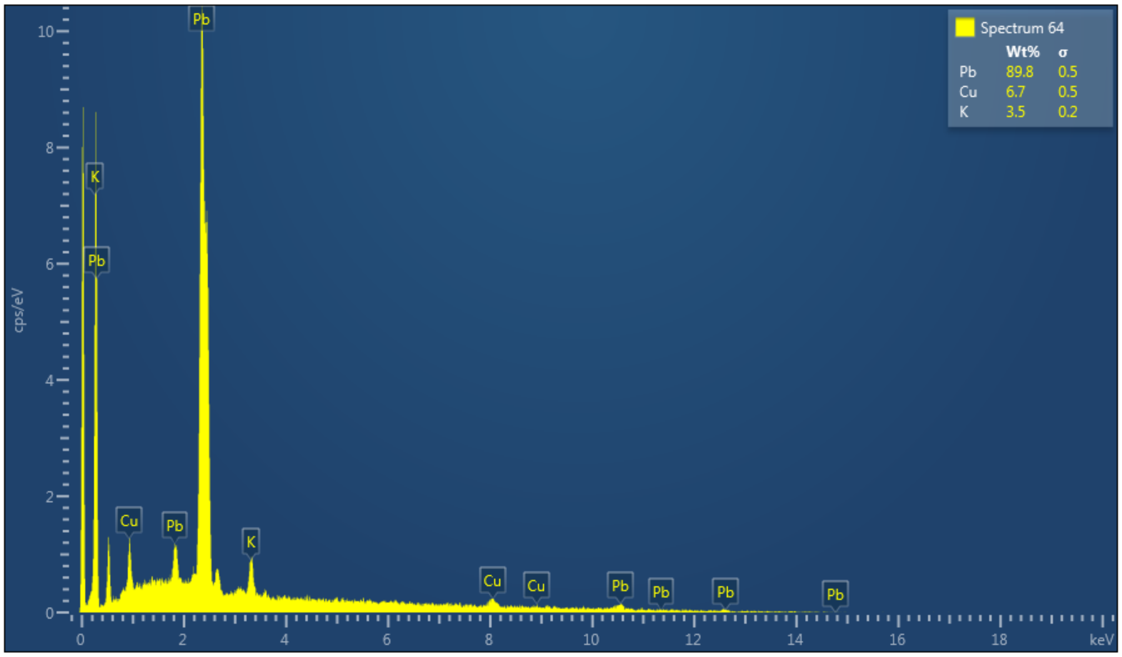

Figure S4: EDX spectrum of $[K(2,2,2{-}crypt)]_4[Cu_4Pb_{22}]$ (K, Cu, Pb).

Table S2: EDX analysis of $[K(2,2,2{-}crypt)]_4[Cu_4Pb_{22}]$.

			Atom %	
Element	wt%	σ	Experimental	Calculated
K	3.5	0.2	14.25	13.33
Cu	6.7	0.5	16.78	13.33
Pb	89.8	0.5	68.97	73.34

Supplementary theoretical data

Bonding of a $[RuCp]^{+}$ fragment to $[CuPb_{11}]^{3-}$

Figure S5 summarizes the key interactions that bind the $[RuCp]^{+}$ fragment to $[CuPb_{11}]^{3-}$. In comparison to the situation with Cu^{+} in Figure 5, the critical difference is that the degenerate HOMO of $[CuPb_{9}]^{3-}$ is involved in the bonding, and is stabilized as a result. The energy decomposition analysis in Table 2 confirms that this is the dominant component of the orbital interaction term.

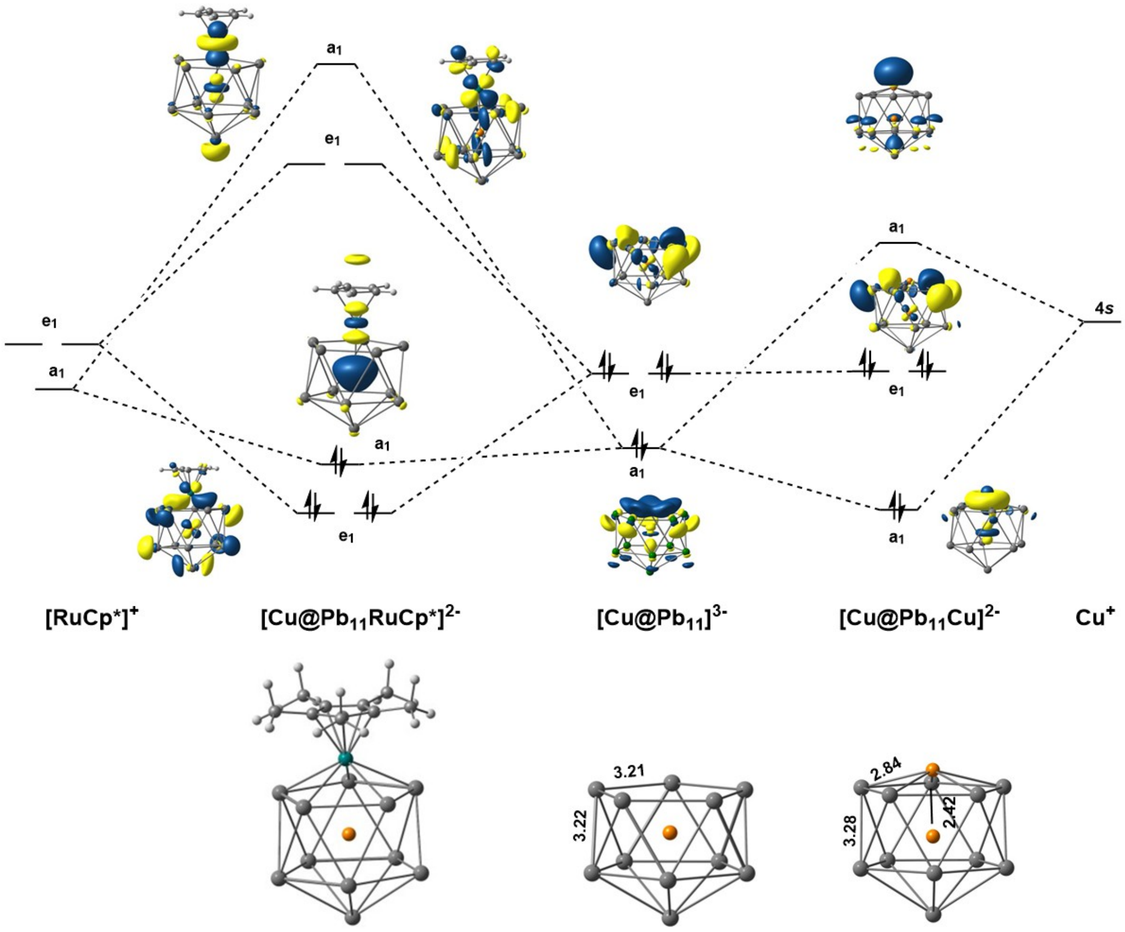

Figure S5: Kohn-Sham MO diagrams for $[Cu_2Pb_{11}]^{2-}$ and $[(Cp^*Ru)CuPb_{11}]^{2-}$ emphasizing the dominant contribution from orbitals of e_1 symmetry.

Comparison to pathway from $[MPb_9]^{3-}$.

The $[CuPb_9]^{3-}$ anion has been synthesized by Fässler and co-workers,[9] and their structural characterization indicates that it adopts a somewhat expanded tri-capped trigonal prism rather than the *nido* mono-capped square antiprism that might be anticipated for a 4n+4 = 40 electron count. At the level of theory used here we find that the most stable local minimum is a C_{4v}-symmetric capped square anti-prism, although the D_{3h}-symmetric alternative is less stable by only 0.19 eV (see supporting information Figure S6). The energetic proximity of these isomers of E_9 clusters has been noted previously (see, for example, Rosdahl *et al.*, *Eur. J. Inorg. Chem.* 2005, 2888–289) and in the present case the discrepancy between computed energies and the X-ray data may well reflect the rather simplistic treatment of the Madelung potential applied in our calculations. Regardless of the identity of the true minimum energy structure, we conclude that the C_{4v}-symmetric *nido* isomer of $[CuPb_9]^{3-}$ is at least thermally accessible in solution, and therefore able to trap a Cu^+ ion on the open face in the same way as $[CuPb_{11}]^{3-}$ does.

Figure S6 summarizes the key interactions that bind the Cu^+ fragment to $[CuPb_9]^{3-}$. The situation is strikingly similar to that shown in Figure 5: the key interaction is of a_1 symmetry, with smaller contributions from e_1.

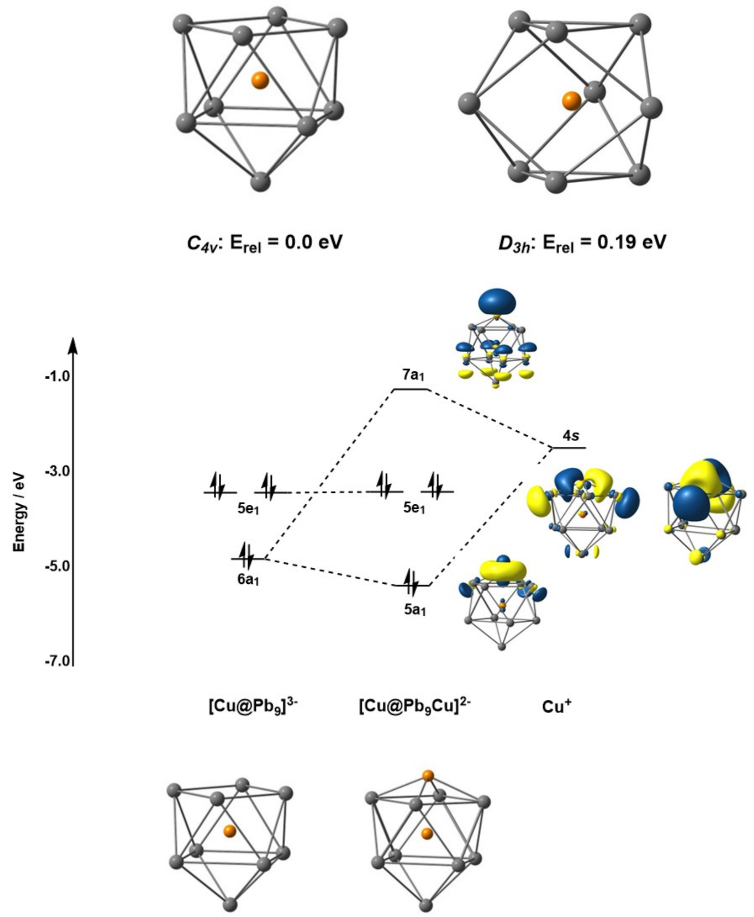

Figure S6: Optimized structures of isomers of $[CuPb_9]^{3-}$ and Kohn-Sham MO diagram for $[Cu_2Pb_9]^{2-}$.

Electronic origins of the isolobal analogy between $[Cu_4Pb_{22}]^{4-}$ and B_2H_6.

Figure S7 highlights the isolobal relationship between $[Cu_4Pb_{22}]^{4-}$ and B_2H_6, as illustrated by the localized orbitals on the bridging region.

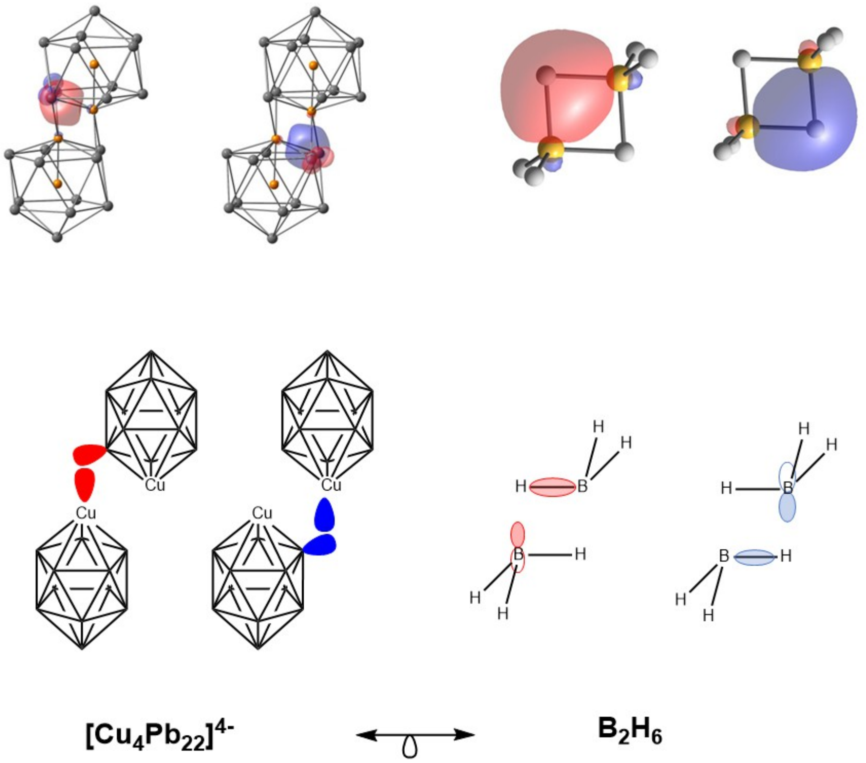

Figure S7: Basis of the isolobal analogy between $[Cu_4Pb_{22}]^{4-}$ and B_2H_6.

Figure S8 compares the structures and energies of two isomers of $[Cu_4Pb_{18}]^{4-}$, and also shows the structure of $[Cu_2Ge_{18}(Mes)_2]^{4-}$. The isomer present in the crystal (D_{2h}) is the more stable by 0.76 eV.

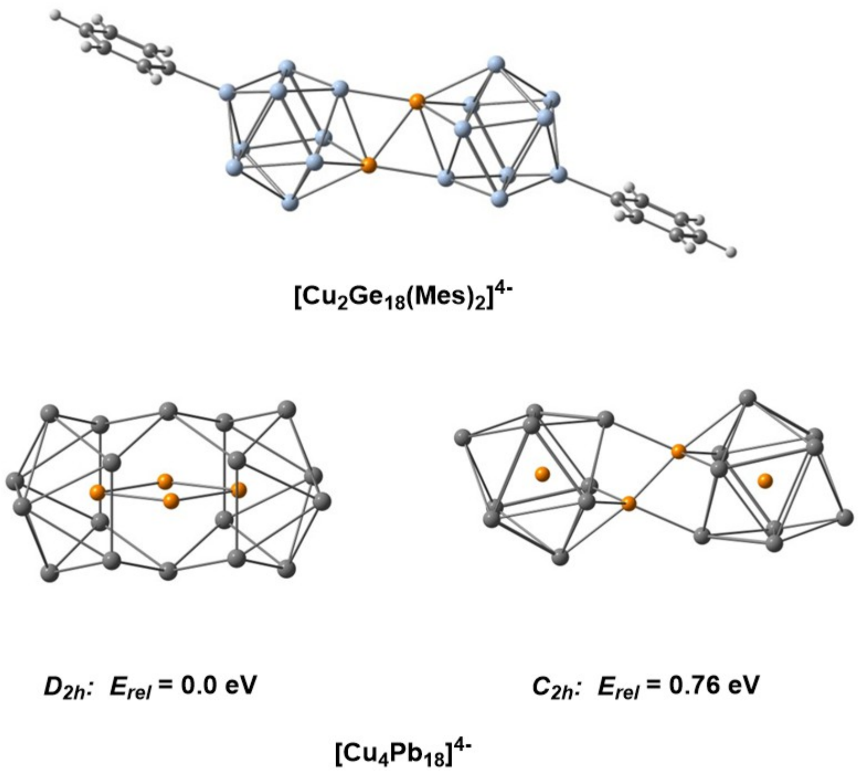

Figure S8: Structure of $[Cu_2Ge_{18}(Mes)_2]^{4-}$ and comparison of the D_{2h}- and C_{2h}-symmetric isomers of $[Cu_4Pb_{18}]^{4-}$.

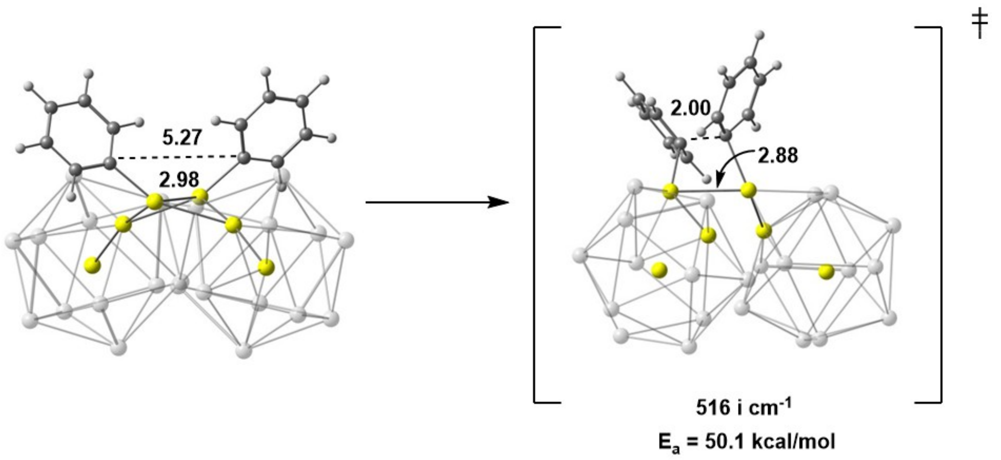

Figure S9: Transition state for C-C coupling of two phenyl units on a Au_6Pb_{22} cluster.

Transition state for C-C coupling.

The optimized structures of the biphenyl intermediate and the ensueing transition state leading to formation of biphenyl, along with two formally zero-valent Au atoms, is shown in Figure S9. The C-C distance of 2.00 Å and conformation of the two phenyl rings is very typical of other such transition states reported in the literature. The barrier is, however, high, at 50.1 kcal/mol, suggesting that this pathway is unlikely to be viable at the temperatures used in the experiments that generate $[Au_8Pb_{33}]^{6-}$ and $[Au_{12}Pb_{44}]^{8-}$.

Energetics of all fundamental steps shown in Figures 4 and 7

.

Table S3: Formation energies of metal atoms and cations, in eV. Values for metal atoms and for Pb are for the $nd^{10}(n+1)s^1$ and $6s^2 6p^2$ configurations, respectively. All energies are given relative to the spin-restricted atomic reference.

	M	M^+
Cu	-0.23	+4.377
Ag	-0.18	+4.184
Au	-0.17	+5.748
Pb	-0.60	

Table S4: Summary of total formation energies of all clusters and fragments. All values in eV

	Cu	Ag	Au
$[MPb_{11}]^{3-}$	-47.667	-46.893	-47.612
$[M_2Pb_{11}]^{2-}$	-48.797	-46.955	-48.403
$[M_2Pb_{11}]^{3-}$	-50.517	-48.894	-50.501
$[M_3Pb_{11}]^{2-}$	-50.722	-48.373	-50.689
$[MPb_9]^{3-}$	-41.327	-39.796	-40.631
$[M_2Pb_9]^{2-}$	-41.795	-39.477	-41.003
$[M_4Pb_{18}]^{4-}$	-85.054	-80.885	-83.750
$[M_4Pb_{22}]^{4-}$	-98.738	-95.021	-97.932
[MPh]	-72.938	-72.142	-72.835
$[PhM_3Pb_{11}]^{2-}$	-123.358	-120.543	-122.946
$[PhM_3Pb_{11}]^{3-}$	-125.488	-122.723	-125.371
$[Ph_2M_6Pb_{22}]^{4-}$	-247.271	-241.618	-245.956
$[M_6Pb_{22}]^{4-}$	-104.255	-99.273	-103.668
$[Ph^-]$	-73.247		
Ph_2	-144.064		

Optimized cartesian coordinates (in Angstrom) and total energies (in eV). Total Energies in ADF are calculated relative to the energies of the isolated spin-restricted atoms) of all minima.

Table S5: $[CuPb_{11}]^{3-}$, E = -47.667 eV

Pb	-0.877104	-2.699449	1.093095
Pb	0.842280	-2.592271	-1.634678
Pb	-2.205118	1.602112	-1.634678
Pb	-2.205118	-1.602112	-1.634678
Pb	2.296288	-1.668351	1.093095
Pb	2.725675	0.000000	-1.634678
Pb	0.842280	2.592271	-1.634678
Pb	2.296288	1.668351	1.093095
Pb	-2.838368	0.000000	1.093095
Pb	0.000000	0.000000	2.791437
Pb	-0.877104	2.699449	1.093095
Cu	0.000000	0.000000	-0.218496

Table S6: $[AgPb_{11}]^{3-}$, E = -46.893 eV

Pb	-0.886453	-2.728222	1.098760
Pb	0.862575	-2.654733	-1.639417
Pb	-2.258250	1.640715	-1.639417
Pb	-2.258250	-1.640715	-1.639417
Pb	2.320764	-1.686134	1.098760
Pb	2.791351	0.000000	-1.639417
Pb	0.862575	2.654733	-1.639417
Pb	2.320764	1.686134	1.098760
Pb	-2.868622	0.000000	1.098760
Pb	0.000000	0.000000	2.829863
Pb	-0.886453	2.728222	1.098760
Ag	0.000000	0.000000	-0.261551

Table S7: $[AuPb_{11}]^{3-}$, E = -47.612 eV

Pb	-0.887232	-2.730620	1.092933
Pb	0.860646	-2.648795	-1.636082
Pb	-2.253200	1.637045	-1.636082
Pb	-2.253200	-1.637045	-1.636082
Pb	2.322804	-1.687616	1.092933
Pb	2.785108	0.000000	-1.636082
Pb	0.860646	2.648795	-1.636082
Pb	2.322804	1.687616	1.092933
Pb	-2.871144	0.000000	1.092933
Pb	0.000000	0.000000	2.823815
Pb	-0.887232	2.730620	1.092933
Au	0.000000	0.000000	-0.243044

Table S8: $[Cu_2Pb_{11}]^{2-}$, E = -48.797 eV

Pb	-0.871389	-2.681861	1.098651
Pb	0.854860	-2.630988	-1.694288
Pb	-2.238052	1.626040	-1.694288
Pb	-2.238052	-1.626040	-1.694288
Pb	2.281327	-1.657481	1.098651
Pb	2.766384	0.000000	-1.694288
Pb	0.854860	2.630988	-1.694288
Pb	2.281327	1.657481	1.098651
Pb	-2.819876	0.000000	1.098651
Pb	0.000000	0.000000	2.899702
Pb	-0.871389	2.681861	1.098651
Cu	0.000000	0.000000	0.096171
Cu	0.000000	0.000000	-2.330396

Table S9: $[Ag_2Pb_{11}]^{2-}$, E = -46.955 eV

Pb	-0.884452	-2.722065	1.082999
Pb	0.877856	-2.701762	-1.692910
Pb	-2.298256	1.669781	-1.692910
Pb	-2.298256	-1.669781	-1.692910
Pb	2.315527	-1.682329	1.082999
Pb	2.840801	0.000000	-1.692910
Pb	0.877856	2.701762	-1.692910
Pb	2.315527	1.682329	1.082999
Pb	-2.862148	0.000000	1.082999
Pb	0.000000	0.000000	2.912411
Pb	-0.884452	2.722065	1.082999
Ag	0.000000	0.000000	-0.022603
Ag	0.000000	0.000000	-2.752963

Table S10: $[Au_2Pb_{11}]^{2-}$, E = -48.403 eV

Pb	-0.887501	-2.731448	1.053804
Pb	0.881009	-2.711468	-1.724832
Pb	-2.306512	1.675779	-1.724832
Pb	-2.306512	-1.675779	-1.724832
Pb	2.323508	-1.688128	1.053804
Pb	2.851006	0.000000	-1.724832
Pb	0.881009	2.711468	-1.724832
Pb	2.323508	1.688128	1.053804
Pb	-2.872014	0.000000	1.053804
Pb	0.000000	0.000000	2.981570
Pb	-0.887501	2.731448	1.053804
Au	0.000000	0.000000	0.107982
Au	0.000000	0.000000	-2.647120

Table S11: $[(Cp^{*}Ru)CuPb_{11}]^{2-}$, E = -116.934 eV

Cu	0.000000	0.000000	-0.011849
Pb	-2.245543	1.631483	-1.373120
Pb	-2.681593	0.000000	1.513349
Pb	-0.828658	2.550347	1.513349
Pb	-2.245543	-1.631483	-1.373120
Pb	0.000000	0.000000	-3.110078
Pb	0.857721	2.639794	-1.373120
Pb	2.169454	-1.576201	1.513349
Pb	2.775644	0.000000	-1.373120
Pb	0.857721	-2.639794	-1.373120
Pb	2.169454	1.576201	1.513349
Pb	-0.828658	-2.550347	1.513349
Ru	0.000000	0.000000	2.829739
C	1.224065	0.000000	4.684350
C	0.378257	-1.164155	4.684350
C	-0.990289	-0.719487	4.684350
C	-0.990289	0.719487	4.684350
C	0.378257	1.164155	4.684350
H	-1.869849	1.358524	4.731377
H	-1.869849	-1.358524	4.731377
H	0.714219	-2.198139	4.731377
H	2.311260	0.000000	4.731377
H	0.714219	2.198139	4.731377

Table S12: $[CuPb_{11}Cu_2]^{2-}$, E = -50.722 eV

Pb	-0.865594	1.086790	2.690375
Pb	0.765738	-1.755200	2.654135
Pb	-2.331758	-1.723391	-1.673534
Pb	-2.331758	-1.723391	1.673534
Pb	2.252614	0.984047	1.664648
Pb	2.694014	-1.805526	0.000000
Pb	0.765738	-1.755200	-2.654135
Pb	2.252614	0.984047	-1.664648
Pb	-2.818725	1.089890	0.000000
Pb	0.025876	2.817403	0.000000
Pb	-0.865594	1.086790	-2.690375
Cu	-0.067989	-0.063590	0.000000
Cu	0.000019	-2.516334	0.000000
Cu	-1.875194	-3.996776	0.000000

Table S13: $[AgPb_{11}Ag_2]^{2-}$, E = -48.373 eV

Pb	-2.752759	0.034625	-1.684378
Pb	-1.867054	-2.646619	0.000000
Pb	2.607525	-0.134640	-1.714058
Pb	-0.153542	-1.659054	-2.725139
Pb	-2.752759	0.034625	1.684378
Pb	-0.153542	-1.659054	2.725139
Pb	2.607525	-0.134640	1.714058
Pb	-0.033104	1.632475	2.722108
Pb	-0.033104	1.632475	-2.722108
Pb	-1.646933	2.737671	0.000000
Pb	1.727406	2.560755	0.000000
Ag	-0.126273	0.133956	0.000000
Ag	1.077558	-2.364283	0.000000
Ag	3.815734	-2.436073	0.000000

Table S14: $[AuPb_{11}Au_2]^{2-}$, E = -50.689 eV

Pb	-0.864678	1.136787	2.724562
Pb	0.822433	-1.703973	2.744240
Pb	-2.326103	-1.702089	-1.724395
Pb	-2.326103	-1.702089	1.724395
Pb	2.283225	1.032155	1.683984
Pb	2.808693	-1.758915	0.000000
Pb	0.822433	-1.703973	-2.744240
Pb	2.283225	1.032155	-1.683984
Pb	-2.864600	1.099904	0.000000
Pb	0.022069	2.937569	0.000000
Pb	-0.864678	1.136787	-2.724562
Au	-0.073169	-0.013428	0.000000
Au	0.106887	-2.799881	0.000000
Au	-2.229634	-4.281450	0.000000

Table S15: $[Cu_4Pb_{22}]^{4-}$, E = -98.738 eV

Pb	0.908616	2.416124	-2.616118
Pb	-1.847138	0.953870	-1.640077
Pb	0.908616	2.416124	2.616118
Pb	-1.847138	0.953870	1.640077
Pb	-1.777338	6.175452	0.000000
Pb	-1.848002	4.203829	2.624155
Pb	-2.608786	-3.131904	0.000000
Pb	-1.848002	4.203829	-2.624155
Pb	-3.567065	3.361187	0.000000
Pb	1.007192	5.550355	1.636630
Pb	1.007192	5.550355	-1.636630
Cu	-0.531773	-1.156194	0.000000
Cu	-0.519603	3.467692	0.000000
Pb	-0.908616	-2.416124	2.616118
Pb	1.847138	-0.953870	1.640077
Pb	-0.908616	-2.416124	-2.616118
Pb	1.847138	-0.953870	-1.640077
Pb	2.608786	3.131904	0.000000
Pb	-1.007192	-5.550355	-1.636630
Pb	-1.007192	-5.550355	1.636630
Cu	0.531773	1.156194	0.000000
Cu	0.519603	-3.467692	0.000000
Pb	1.777338	-6.175452	0.000000
Pb	1.848002	-4.203829	-2.624155
Pb	1.848002	-4.203829	2.624155
Pb	3.567065	-3.361187	0.000000

Table S16: $[Ag_4Pb_{22}]^{4-}$, E = -95.021 eV

Pb	0.963782	2.674573	-2.683472
Pb	-1.851295	1.186765	-1.697940
Pb	0.963782	2.674573	2.683472
Pb	-1.851295	1.186765	1.697940
Pb	-1.872697	6.373063	0.000000
Pb	-1.872971	4.386310	2.684199
Pb	-2.705194	-3.528356	0.000000
Pb	-1.872971	4.386310	-2.684199
Pb	-3.615204	3.488933	0.000000
Pb	0.979588	5.810733	1.670309
Pb	0.979588	5.810733	-1.670309
Ag	-0.804648	-1.172099	0.000000
Ag	-0.522188	3.650843	0.000000
Pb	-0.963782	-2.674573	2.683472
Pb	1.851295	-1.186765	1.697940
Pb	-0.963782	-2.674573	-2.683472
Pb	1.851295	-1.186765	-1.697940
Pb	2.705194	3.528356	0.000000
Pb	-0.979588	-5.810733	-1.670309
Pb	-0.979588	-5.810733	1.670309
Ag	0.804648	1.172099	0.000000
Ag	0.522188	-3.650843	0.000000
Pb	1.872697	-6.373063	0.000000
Pb	1.872971	-4.386310	-2.684199
Pb	1.872971	-4.386310	2.684199
Pb	3.615204	-3.488933	0.000000

Table S17: $[Au_4Pb_{22}]^{4-}$, E = -97.932 eV

Pb	0.960787	2.636562	-2.681851
Pb	-1.852733	1.112523	-1.699212
Pb	0.960787	2.636562	2.681851
Pb	-1.852733	1.112523	1.699212
Pb	-1.859984	6.373134	0.000000
Pb	-1.871531	4.366211	2.674058
Pb	-2.717444	-3.450164	0.000000
Pb	-1.871531	4.366211	-2.674058
Pb	-3.616514	3.462526	0.000000
Pb	0.992827	5.775919	1.666583
Pb	0.992827	5.775919	-1.666583
Au	-0.783996	-1.193435	0.000000
Au	-0.560362	3.679545	0.000000
Pb	-0.960787	-2.636562	2.681851
Pb	1.852733	-1.112523	1.699212
Pb	-0.960787	-2.636562	-2.681851
Pb	1.852733	-1.112523	-1.699212
Pb	2.717444	3.450164	0.000000
Pb	-0.992827	-5.775919	-1.666583
Pb	-0.992827	-5.775919	1.666583
Au	0.783996	1.193435	0.000000
Au	0.560362	-3.679545	0.000000
Pb	1.859984	-6.373134	0.000000
Pb	1.871531	-4.366211	-2.674058
Pb	1.871531	-4.366211	2.674058
Pb	3.616514	-3.462526	0.000000

Table S18: D_{2h} isomer of $[Cu_4Pb_{18}]^{4-}$, E = -85.054 eV

Pb	-2.525697	1.990615	1.599481
Pb	-2.525697	1.990615	-1.599481
Pb	-2.525697	-1.990615	-1.599481
Pb	-2.525697	-1.990615	1.599481
Pb	0.000000	0.000000	-2.619514
Pb	0.000000	3.783001	-2.609268
Pb	0.000000	3.783001	2.609268
Pb	1.671526	4.773158	0.000000
Pb	-1.671526	4.773158	0.000000
Cu	-1.278727	0.000000	0.000000
Cu	0.000000	2.252639	0.000000
Pb	2.525697	-1.990615	-1.599481
Pb	2.525697	-1.990615	1.599481
Pb	2.525697	1.990615	1.599481
Pb	2.525697	1.990615	-1.599481
Pb	0.000000	0.000000	2.619514
Pb	0.000000	-3.783001	2.609268
Pb	0.000000	-3.783001	-2.609268
Pb	-1.671526	-4.773158	0.000000
Pb	1.671526	-4.773158	0.000000
Cu	1.278727	0.000000	0.000000
Cu	0.000000	-2.252639	0.000000

Table S19: C_{2h} isomer of $[Cu_4Pb_{18}]^{4-}$, E = -84.293 eV

Pb	-0.712626	2.145744	2.448169
Pb	1.374027	4.522264	1.863356
Pb	1.793448	1.845216	0.000000
Pb	-1.793448	-1.845216	0.000000
Pb	0.712626	-2.145744	-2.448169
Pb	-0.016343	6.715839	0.000000
Pb	-3.125178	2.519797	0.000000
Pb	1.374027	4.522264	-1.863356
Pb	-1.374027	-4.522264	1.863356
Pb	-0.712626	2.145744	-2.448169
Pb	-1.374027	-4.522264	-1.863356
Pb	0.016343	-6.715839	0.000000
Pb	-2.091078	5.051056	-1.779144
Pb	-2.091078	5.051056	1.779144
Pb	2.091078	-5.051056	1.779144
Pb	2.091078	-5.051056	-1.779144
Pb	3.125178	-2.519797	0.000000
Pb	0.712626	-2.145744	2.448169
Cu	-0.877894	0.868344	0.000000
Cu	0.877894	-0.868344	0.000000
Cu	-0.483142	3.586637	0.000000
Cu	0.483142	-3.586637	0.000000

Table S20: $[Ag_4Pb_{18}]^{4-}$, E = -80.885 eV

Pb	-2.575851	2.217766	1.653212
Pb	-2.575851	2.217766	-1.653212
Pb	-2.575851	-2.217766	-1.653212
Pb	-2.575851	-2.217766	1.653212
Pb	0.000000	0.000000	-2.654202
Pb	0.000000	3.987056	-2.654580
Pb	0.000000	3.987056	2.654580
Pb	1.698527	4.983866	0.000000
Pb	-1.698527	4.983866	0.000000
Ag	-1.509191	0.000000	0.000000
Ag	0.000000	2.440067	0.000000
Pb	2.575851	-2.217766	-1.653212
Pb	2.575851	-2.217766	1.653212
Pb	2.575851	2.217766	1.653212
Pb	2.575851	2.217766	-1.653212
Pb	0.000000	0.000000	2.654202
Pb	0.000000	-3.987056	2.654580
Pb	0.000000	-3.987056	-2.654580
Pb	-1.698527	-4.983866	0.000000
Pb	1.698527	-4.983866	0.000000
Ag	1.509191	0.000000	0.000000
Ag	0.000000	-2.440067	0.000000

Table S21: $[Au_4Pb_{18}]^{4-}$, E = -83.750 eV

Pb	-2.598771	2.201501	1.658271
Pb	-2.598771	2.201501	-1.658271
Pb	-2.598771	-2.201501	-1.658271
Pb	-2.598771	-2.201501	1.658271
Pb	0.000000	0.000000	-2.625270
Pb	0.000000	3.961675	-2.654477
Pb	0.000000	3.961675	2.654477
Pb	1.714218	4.973905	0.000000
Pb	-1.714218	4.973905	0.000000
Au	-1.543847	0.000000	0.000000
Au	0.000000	2.487070	0.000000
Pb	2.598771	-2.201501	-1.658271
Pb	2.598771	-2.201501	1.658271
Pb	2.598771	2.201501	1.658271
Pb	2.598771	2.201501	-1.658271
Pb	0.000000	0.000000	2.625270
Pb	0.000000	-3.961675	2.654477
Pb	0.000000	-3.961675	-2.654477
Pb	-1.714218	-4.973905	0.000000
Pb	1.714218	-4.973905	0.000000
Au	1.543847	0.000000	0.000000
Au	0.000000	-2.487070	0.000000

Table S22: $[CpRu]^{+}$, E = -63.997 eV

Ru	0.000000	0.000000	3.003154
C	1.228777	0.000000	4.706675
C	0.379713	-1.168636	4.706675
C	-0.994101	-0.722257	4.706675
C	-0.994101	0.722257	4.706675
C	0.379713	1.168636	4.706675
H	-1.873357	1.361073	4.667540
H	-1.873357	-1.361073	4.667540
H	0.715559	-2.202263	4.667540
H	2.315596	0.000000	4.667540
H	0.715559	2.202263	4.667540

Table S23: $[CuPb_9]^{3-}$, E = -41.327 eV

Cu	0.000000	0.000000	0.239377
Pb	0.000000	0.000000	3.230545
Pb	-1.810001	-1.810001	1.304923
Pb	-1.810001	1.810001	1.304923
Pb	1.810001	1.810001	1.304923
Pb	1.810001	-1.810001	1.304923
Pb	0.000000	2.316097	-1.360894
Pb	-2.316097	0.000000	-1.360894
Pb	0.000000	-2.316097	-1.360894
Pb	2.316097	0.000000	-1.360894

Table S24: $[AgPb_9]^{3-}$, E = -39.796 eV

Ag	0.000000	0.000000	0.236494
Pb	0.000000	0.000000	3.250366
Pb	-1.870623	-1.870623	1.316800
Pb	-1.870623	1.870623	1.316800
Pb	1.870623	1.870623	1.316800
Pb	1.870623	-1.870623	1.316800
Pb	0.000000	2.395241	-1.377005
Pb	-2.395241	0.000000	-1.377005
Pb	0.000000	-2.395241	-1.377005
Pb	2.395241	0.000000	-1.377005

Table S25: $[AuPb_9]^{3-}$, E = -40.631 eV

Au	0.000000	0.000000	0.247318
Pb	0.000000	0.000000	3.251144
Pb	-1.883037	-1.883037	1.319761
Pb	-1.883037	1.883037	1.319761
Pb	1.883037	1.883037	1.319761
Pb	1.883037	-1.883037	1.319761
Pb	0.000000	2.397420	-1.382867
Pb	-2.397420	0.000000	-1.382867
Pb	0.000000	-2.397420	-1.382867
Pb	2.397420	0.000000	-1.382867

Table S26: $[Cu_2Pb_9]^{3-}$, E = -41.795 eV

Cu	0.000000	0.000000	-2.339679
Cu	0.000000	0.000000	0.226849
Pb	0.000000	0.000000	3.254058
Pb	-1.800758	-1.800758	1.318715
Pb	-1.800758	1.800758	1.318715
Pb	1.800758	1.800758	1.318715
Pb	1.800758	-1.800758	1.318715
Pb	0.000000	2.501815	-1.292512
Pb	-2.501815	0.000000	-1.292512
Pb	0.000000	-2.501815	-1.292512
Pb	2.501815	0.000000	-1.292512

Table S27: $[Ag_2gPb_9]^{3-}$, E = -41.003 eV

Ag	0.000000	0.000000	-2.684638
Ag	0.000000	0.000000	0.217607
Pb	0.000000	0.000000	3.284866
Pb	-1.859102	-1.859102	1.367591
Pb	-1.859102	1.859102	1.367591
Pb	1.859102	1.859102	1.367591
Pb	1.859102	-1.859102	1.367591
Pb	0.000000	2.582799	-1.260540
Pb	-2.582799	0.000000	-1.260540
Pb	0.000000	-2.582799	-1.260540
Pb	2.582799	0.000000	-1.260540

Table S28: $[Au_2Pb_9]^{3-}$, E = -39.477 eV

Au	0.000000	0.000000	-2.668340
Au	0.000000	0.000000	0.288914
Pb	0.000000	0.000000	3.297287
Pb	-1.878908	-1.878908	1.360610
Pb	-1.878908	1.878908	1.360610
Pb	1.878908	1.878908	1.360610
Pb	1.878908	-1.878908	1.360610
Pb	0.000000	2.577470	-1.278565
Pb	-2.577470	0.000000	-1.278565
Pb	0.000000	-2.577470	-1.278565
Pb	2.577470	0.000000	-1.278565

Table S29: [PhCu, E = -72.938eV

Cu	-0.498663	2.275392	-3.903342
C	-0.612073	2.873234	-5.654417
C	0.547933	3.116665	-6.422750
C	-1.862165	3.103365	-6.269644
C	0.460994	3.569364	-7.746176
C	-1.948958	3.556022	-7.593334
C	-0.787668	3.791267	-8.336689
H	1.375812	3.748733	-8.317073
H	-2.930656	3.725918	-8.043155
H	-2.789069	2.930494	-5.717891
H	1.538964	2.953706	-5.992957
H	-0.854777	4.144531	-9.367991

Table S30: [PhAg, E = -72.142

Ag	-0.489414	2.197888	-3.718292
C	-0.612820	2.883355	-5.674186
C	0.545836	3.125983	-6.438036
C	-1.861328	3.112347	-6.285136
C	0.459281	3.576256	-7.763572
C	-1.949278	3.562561	-7.610523
C	-0.788755	3.795334	-8.355230
H	1.374843	3.754522	-8.334055
H	-2.931761	3.730844	-8.059726
H	-2.787402	2.940884	-5.731310
H	1.536814	2.964572	-6.007061
H	-0.856343	4.144145	-9.388292

Table S31: [PhAu, E = -72.835eV

Au	-0.493155	2.243963	-3.813842
C	-0.614480	2.886604	-5.692626
C	0.555817	3.119691	-6.431750
C	-1.871199	3.106492	-6.277747
C	0.461401	3.572285	-7.754498
C	-1.950475	3.559155	-7.601418
C	-0.787874	3.792780	-8.341755
H	1.376252	3.751606	-8.323820
H	-2.931937	3.728080	-8.050168
H	-2.788129	2.929483	-5.715109
H	1.538568	2.953151	-5.990058
H	-0.855117	4.145403	-9.372629

Table S32: [Ph_2, E = -144.064eV

C	-0.668777	2.782428	-5.285432
C	-0.324333	1.890187	-6.318933
C	-1.043409	4.093879	-5.635963
C	-0.353087	2.295081	-7.655226
C	-1.073098	4.498569	-6.972351
C	-0.727660	3.601153	-7.988472
H	-0.073755	1.589274	-8.440211
H	-1.374445	5.518031	-7.222036
H	-1.336092	4.796524	-4.853135
H	-0.009137	0.874882	-6.069839
H	-0.750144	3.917365	-9.033150
C	-0.635998	2.352190	-3.864365
C	-1.003292	1.043444	-3.496430
C	-0.235688	3.241457	-2.848587
C	-0.970584	0.638136	-2.160309
C	-0.202445	2.836046	-1.512537
C	-0.569752	1.532431	-1.161956
H	-1.268104	-0.378860	-1.896426
H	0.119439	3.539444	-0.741843
H	0.074619	4.254471	-3.112607
H	-1.338947	0.343378	-4.264206
H	-0.543799	1.215643	-0.117559

Table S33: [Ph^-, E = -73.247eV

C	-0.664591	2.762950	-5.217533
C	0.143854	2.183678	-6.234109
C	-1.507604	3.798033	-5.707734
C	0.126126	2.577592	-7.584167
C	-1.553263	4.217534	-7.049525
C	-0.730163	3.604073	-8.002119
H	0.779929	2.084752	-8.312683
H	-2.229799	5.024038	-7.354350
H	-2.181169	4.322866	-5.014698
H	0.838227	1.370301	-5.977577
H	-0.754169	3.920200	-9.047986

Table S34: $[Cu_2Pb_{11}]^{3-}$, E = -50.517 eV

Pb	-0.873422	-2.688117	1.107640
Pb	0.842697	-2.593554	-1.721468
Pb	-2.206209	1.602905	-1.721468
Pb	-2.206209	-1.602905	-1.721468
Pb	2.286649	-1.661347	1.107640
Pb	2.727025	0.000000	-1.721468
Pb	0.842697	2.593554	-1.721468
Pb	2.286649	1.661347	1.107640
Pb	-2.826453	0.000000	1.107640
Pb	0.000000	0.000000	2.832736
Pb	-0.873422	2.688117	1.107640
Cu	0.000000	0.000000	-0.076981
Cu	0.000000	0.000000	-2.599324

Table S35: $[Ag_2Pb_{11}]^{3-}$, E = -48.894 eV

Pb	-0.883195	-2.718196	1.135019
Pb	0.863959	-2.658992	-1.679311
Pb	-2.261873	1.643347	-1.679311
Pb	-2.261873	-1.643347	-1.679311
Pb	2.312236	-1.679938	1.135019
Pb	2.795829	0.000000	-1.679311
Pb	0.863959	2.658992	-1.679311
Pb	2.312236	1.679938	1.135019
Pb	-2.858081	0.000000	1.135019
Pb	0.000000	0.000000	2.901883
Pb	-0.883195	2.718196	1.135019
Ag	0.000000	0.000000	-0.117688
Ag	0.000000	0.000000	-2.975448

Table S36: $[Au_2Pb_{11}]^{3-}$, E = -48.894 eV

Pb	-0.884394	-2.721883	1.123530
Pb	0.864302	-2.660048	-1.697215
Pb	-2.262772	1.644000	-1.697215
Pb	-2.262772	-1.644000	-1.697215
Pb	2.315372	-1.682216	1.123530
Pb	2.796940	0.000000	-1.697215
Pb	0.864302	2.660048	-1.697215
Pb	2.315372	1.682216	1.123530
Pb	-2.861958	0.000000	1.123530
Pb	0.000000	0.000000	2.915522
Pb	-0.884394	2.721883	1.123530
Au	0.000000	0.000000	-0.039443
Au	0.000000	0.000000	-2.920365

Table S37: $[Cu_6Pb_{22}]^{4-}$, E = -104.255 eV

Cu	0.045650	-1.235888	0.123632
Cu	1.953431	0.072583	0.791518
Cu	-0.045650	1.235888	0.123632
Cu	4.255422	-0.052500	-0.190263
Cu	-4.255422	0.052500	-0.190263
Cu	-1.953431	-0.072583	0.791518
Pb	3.676929	-0.963623	2.808566
Pb	2.268262	2.346026	-1.112458
Pb	2.572832	-2.718294	0.282686
Pb	-3.423060	-2.146390	1.967057
Pb	1.754241	-0.699494	-2.186253
Pb	3.423060	2.146390	1.967057
Pb	-3.676929	0.963623	2.808566
Pb	-2.268262	-2.346026	-1.112458
Pb	5.857519	-2.424033	0.871168
Pb	-6.392440	-0.642427	1.935227
Pb	4.497384	0.898943	-3.077386
Pb	-7.022529	0.141430	-1.190042
Pb	7.022529	-0.141430	-1.190042
Pb	4.688230	-2.259455	-2.218756
Pb	-5.857519	2.424033	0.871168
Pb	-1.754241	0.699494	-2.186253
Pb	6.392440	0.642427	1.935227
Pb	5.491470	2.708813	-0.488228
Pb	-5.491470	-2.708813	-0.488228
Pb	-4.688230	2.259455	-2.218756
Pb	-2.572832	2.718294	0.282686
Pb	-4.497384	-0.898943	-3.077386

Table S38: $[Ag_6Pb_{22}]^{4-}$, E = -99.273 eV

Ag	0.021134	-1.445674	0.036134
Ag	2.008640	0.036940	1.220811
Ag	-0.021134	1.445674	0.036134
Ag	4.468392	0.049437	-0.195780
Ag	-4.468392	-0.049437	-0.195780
Ag	-2.008640	-0.036940	1.220811
Pb	4.140025	-1.487902	2.649911
Pb	2.692905	2.696226	-0.520871
Pb	2.830148	-2.694222	-0.225517
Pb	-4.037574	-1.833292	2.464372
Pb	1.955397	-0.118983	-2.192003
Pb	4.037574	1.833292	2.464372
Pb	-4.140025	1.487902	2.649911
Pb	-2.692905	-2.696226	-0.520871
Pb	6.099221	-2.586300	0.252996
Pb	-6.848670	-0.216374	1.897554
Pb	4.719077	1.571704	-2.909429
Pb	-7.295658	-0.057757	-1.409065
Pb	7.295658	0.057757	-1.409065
Pb	4.809625	-1.737632	-2.727116
Pb	-6.099221	2.586300	0.252996
Pb	-1.955397	0.118983	-2.192003
Pb	6.848670	0.216374	1.897554
Pb	5.944723	2.789223	-0.025531
Pb	-5.944723	-2.789223	-0.025531
Pb	-4.809625	1.737632	-2.727116
Pb	-2.830148	2.694222	-0.225517
Pb	-4.719077	-1.571704	-2.909429

Table S39: $[Au_6Pb_{22}]^{4-}$, E = -103.668 eV

Au	0.096146	-1.481244	0.608640
Au	2.172982	0.166188	1.413987
Au	-0.096146	1.481244	0.608640
Au	4.446197	-0.045922	-0.304157
Au	-4.446197	0.045922	-0.304157
Au	-2.172982	-0.166188	1.413987
Pb	4.450723	-0.983481	2.894974
Pb	2.380553	2.406450	-0.866795
Pb	2.864652	-2.756233	0.483819
Pb	-4.107154	-2.232192	2.096198
Pb	1.704981	-0.676558	-1.913597
Pb	4.107154	2.232192	2.096198
Pb	-4.450723	0.983481	2.894974
Pb	-2.380553	-2.406450	-0.866795
Pb	6.207469	-2.448928	0.521010
Pb	-6.952027	-0.624040	1.494524
Pb	4.307007	0.926121	-3.216437
Pb	-7.111516	0.154408	-1.736337
Pb	7.111516	-0.154408	-1.736337
Pb	4.577626	-2.279397	-2.399771
Pb	-6.207469	2.448928	0.521010
Pb	-1.704981	0.676558	-1.913597
Pb	6.952027	0.624040	1.494524
Pb	5.682226	2.734640	-0.759590
Pb	-5.682226	-2.734640	-0.759590
Pb	-4.577626	2.279397	-2.399771
Pb	-2.864652	2.756233	0.483819
Pb	-4.307007	-0.926121	-3.216437

Table S40: $[Cu_6Pb_{22}Ph_2]^{4-}$, E = -247.271 eV

Cu	0.714037	-1.233705	1.779231
Cu	2.345753	0.829510	1.576364
Cu	-0.119971	1.028470	1.925583
Cu	4.498667	0.318496	0.139783
Cu	-4.011875	-0.482107	0.475619
Cu	-1.759216	-1.048164	1.694648
Pb	4.840920	0.659540	3.188544
Pb	2.277053	2.118401	-1.111184
Pb	3.421506	-1.974578	2.002960
Pb	-3.549568	-3.366542	1.423163
Pb	1.768329	-1.060311	-0.776585
Pb	4.145136	3.138295	1.294475
Pb	-4.117476	-1.021944	3.513686
Pb	-1.863870	-2.169765	-1.045104
Pb	6.670438	-1.442558	1.503082
Pb	-6.553388	-2.013152	1.511617
Pb	4.072938	0.043653	-2.897261
Pb	-6.680642	0.142731	-0.955453
Pb	7.053032	-0.241609	-1.521290
Pb	4.743702	-2.539071	-0.938486
Pb	-6.082810	1.157409	2.103733
Pb	-1.356815	0.992150	-0.553579
Pb	7.133474	1.756990	1.075536
Pb	5.527488	2.668328	-1.640089
Pb	-5.143617	-2.733367	-1.366864
Pb	-4.352472	2.429507	-0.411402
Pb	-2.812469	1.700457	2.385812
Pb	-3.806997	-0.017159	-2.564555
C	-0.507651	-2.205284	3.027360
C	-0.348862	-3.607450	2.842536
C	-0.565641	-1.774227	4.380901
C	-0.268127	-4.505549	3.913488
C	-0.503197	-2.664183	5.457804
C	-0.353564	-4.039844	5.231406
H	-0.293720	-4.004188	1.824257
H	-0.673277	-0.706987	4.588942
H	-0.142129	-5.574993	3.718936
H	-0.569046	-2.286204	6.482757
H	-0.299668	-4.737096	6.071347
C	1.244596	1.922869	3.076152
C	1.394185	1.420220	4.398383
C	1.082377	3.333654	2.975766
C	1.412243	2.252997	5.521821
C	1.085350	4.174395	4.095202
C	1.257474	3.638914	5.378069
H	1.510007	0.343403	4.541849
H	0.958756	3.783313	1.986169
H	1.545914	1.820251	6.518132
H	[illegible]	[illegible]	[illegible]

Table S41: $[Ag_6Pb_{22}Ph_2]^{4-}$, E = -241.618 eV

Ag	0.924564	-1.429515	1.999436
Ag	2.483897	1.042098	1.620535
Ag	-0.277788	1.184148	2.120016
Ag	4.653046	0.533733	-0.168707
Ag	-4.165927	-0.660750	0.150069
Ag	-1.867634	-1.260856	1.732452
Pb	5.325748	0.993197	2.909757
Pb	2.183667	2.256969	-1.266876
Pb	3.887424	-1.811061	1.881715
Pb	-3.716791	-3.647710	1.103377
Pb	1.841261	-1.002549	-0.798355
Pb	4.288925	3.459101	0.989244
Pb	-4.600057	-1.303397	3.237715
Pb	-1.789664	-2.304828	-1.238097
Pb	7.021430	-1.102277	1.057559
Pb	-6.736654	-2.321868	0.935020
Pb	3.875112	0.178531	-3.163946
Pb	-6.688190	-0.078798	-1.525553
Pb	7.033371	0.048752	-2.067363
Pb	4.861219	-2.372531	-1.202732
Pb	-6.432257	0.889544	1.648553
Pb	-1.414104	0.924789	-0.608401
Pb	7.282386	2.145466	0.509860
Pb	5.343274	2.931473	-2.084473
Pb	-5.006586	-2.945260	-1.839760
Pb	-4.455393	2.297672	-0.693246
Pb	-3.243733	1.567859	2.267830
Pb	-3.627054	-0.129168	-2.870520
C	-0.474321	-2.484307	3.347760
C	-0.533783	-3.892752	3.207340
C	-0.664330	-1.986100	4.659524
C	-0.774339	-4.745180	4.292395
C	-0.912809	-2.827213	5.752289
C	-0.970054	-4.214617	5.573557
H	-0.402593	-4.337902	2.216549
H	-0.630715	-0.906554	4.832633
H	-0.813908	-5.827758	4.137822
H	-1.064447	-2.398502	6.747608
H	-1.163138	-4.874532	6.423020
C	1.224222	2.169098	3.408740
C	1.525722	1.594339	4.667220
C	1.253297	3.584204	3.351956
C	1.846559	2.371294	5.788011
C	1.563438	4.373103	4.467170
C	1.866983	3.768183	5.693228
H	1.520766	0.505727	4.772550
H	1.039000	4.088142	2.404691
H	2.082700	1.886844	6.740401
H	[illegible]	[illegible]	[illegible]

Table S42: $[Au_6Pb_{22}Ph_2]^{4-}$, E = -245.956 eV

Au	0.877837	-1.545573	2.152148
Au	2.433911	0.710545	1.272892
Au	-0.212842	1.234890	2.292210
Au	4.762707	0.436293	-0.281415
Au	-4.282503	-0.559622	0.040124
Au	-1.831435	-0.970831	1.368554
Pb	5.075269	0.890461	2.903670
Pb	2.084912	2.100456	-1.431309
Pb	3.814049	-1.965425	1.835002
Pb	-3.410315	-3.519648	0.976157
Pb	1.724252	-1.194503	-0.865235
Pb	3.985822	3.291804	0.975718
Pb	-4.346885	-1.304084	3.183476
Pb	-1.699842	-2.108676	-1.461576
Pb	7.002057	-1.105869	1.112706
Pb	-6.575154	-2.374772	0.954425
Pb	3.922370	0.043830	-3.182712
Pb	-6.765944	-0.039268	-1.481981
Pb	7.116444	0.047813	-2.028148
Pb	4.929686	-2.448070	-1.205972
Pb	-6.410373	0.832755	1.732227
Pb	-1.316684	1.140145	-0.638792
Pb	7.130759	2.157256	0.608956
Pb	5.277608	2.872384	-2.066669
Pb	-4.930157	-2.835075	-1.905358
Pb	-4.529117	2.394263	-0.611829
Pb	-3.168571	1.639432	2.280880
Pb	-3.668471	0.092743	-2.871042
C	-0.105117	-2.755178	3.533954
C	-0.251381	-4.146395	3.347020
C	-0.570860	-2.225302	4.755839
C	-0.815371	-4.966384	4.333294
C	-1.135553	-3.038650	5.747480
C	-1.259142	-4.417349	5.542481
H	0.076740	-4.604691	2.410246
H	-0.503378	-1.149231	4.935599
H	-0.912117	-6.041875	4.156246
H	-1.486262	-2.589692	6.681582
H	-1.699942	-5.056552	6.311399
C	0.855814	2.403434	3.648871
C	1.453977	1.841058	4.796281
C	0.944076	3.804362	3.507496
C	2.092167	2.633370	5.759690
C	1.580650	4.604665	4.466069
C	2.158317	4.022566	5.600514
H	1.433114	0.757143	4.936078
H	0.512056	4.287758	2.626979
H	2.547074	2.163292	6.636681
H	[illegible]	[illegible]	[illegible]

Table S43: $[PhCu_3Pb_{11}]^{2-}$, E = -123.358 eV

Pb	1.133172	-2.694830	0.359120
Pb	2.693936	-0.831393	-1.836178
Pb	-2.445707	0.488993	-2.041722
Pb	-0.299978	-1.938667	-2.506018
Pb	2.855215	-0.038924	1.338097
Pb	2.445475	2.295444	-0.945678
Pb	-0.704084	3.172133	-1.052764
Pb	0.736792	2.431734	1.823804
Pb	-2.050460	-1.863731	0.238633
Pb	-0.133519	-0.670352	2.700524
Pb	-2.329236	1.332327	1.132405
Cu	0.157286	0.136274	-0.001362
Cu	0.446053	0.803268	-2.324845
Cu	-0.964260	2.236445	-3.731389
C	-0.881674	2.814898	-5.561940
C	-0.029823	2.173623	-6.497125
C	-1.647832	3.891791	-6.076218
C	0.055977	2.571295	-7.838940
C	-1.572821	4.300189	-7.415838
C	-0.717192	3.640798	-8.306614
H	0.730409	2.046725	-8.522161
H	-2.184850	5.136071	-7.767440
H	-2.328180	4.437065	-5.412847
H	0.591457	1.332289	-6.170971
H	-0.654914	3.955976	-9.351150

Table S44: $[PhAg_3Pb_{11}]^{2-}$, E = -120.543 eV

Pb	0.249134	-3.079815	1.150844
Pb	2.343587	-1.991941	-1.137047
Pb	-2.388964	0.591979	-1.835819
Pb	-0.889309	-2.409225	-1.868052
Pb	2.599869	-0.810839	1.920494
Pb	2.909975	1.260821	-0.622906
Pb	0.028067	2.929256	-1.037804
Pb	1.167932	2.196815	1.974065
Pb	-2.643584	-1.482338	0.722404
Pb	-0.535747	-0.470429	3.170384
Pb	-2.108966	1.828161	1.197792
Ag	0.071201	-0.123498	0.293572
Ag	0.650288	0.178062	-2.366035
Ag	-0.738990	2.133635	-3.851029
C	-0.751239	2.900861	-5.845129
C	0.404895	2.878245	-6.660425
C	-1.901845	3.480900	-6.429449
C	0.418137	3.395835	-7.964506
C	-1.903870	4.004709	-7.731114
C	-0.739320	3.965651	-8.506527
H	1.336116	3.356633	-8.558550
H	-2.817545	4.445136	-8.141645
H	-2.834997	3.531646	-5.858191
H	1.333913	2.444578	-6.274836
H	-0.735146	4.371944	-9.520920

Table S45: $[PhAu_3Pb_{11}]^{2-}$, E = -122.946 eV

Pb	0.393611	-3.064865	1.147630
Pb	2.536872	-1.784520	-1.019993
Pb	-2.297664	0.462860	-2.037572
Pb	-0.602737	-2.437653	-1.948448
Pb	2.529601	-0.638987	2.082567
Pb	2.852164	1.487070	-0.437317
Pb	-0.098147	2.960666	-1.070290
Pb	0.887301	2.262934	2.050100
Pb	-2.570938	-1.662509	0.534029
Pb	-0.713941	-0.543900	3.188499
Pb	-2.298263	1.683268	1.030429
Au	0.030970	-0.130281	0.418086
Au	0.738914	0.262176	-2.226608
Au	-0.522214	2.054141	-3.963551
C	-0.652406	2.870525	-5.868909
C	0.311010	2.582046	-6.863406
C	-1.708888	3.730816	-6.247567
C	0.225209	3.115199	-8.156666
C	-1.801756	4.268119	-7.538874
C	-0.834152	3.961934	-8.502160
H	0.990133	2.866902	-8.897571
H	-2.635668	4.928125	-7.792887
H	-2.484712	3.990629	-5.521043
H	1.153602	1.924640	-6.628508
H	-0.904309	4.377448	-9.510397

Table S46: $[PhCu_3Pb_{11}]^{3-}$, E = -125.488 eV

Pb	1.667628	-2.418828	0.980933
Pb	3.256340	-0.195504	-0.859970
Pb	-1.685588	-0.202899	-2.596192
Pb	0.978436	-2.004795	-2.234311
Pb	2.333058	0.593676	2.204325
Pb	2.023233	2.728365	-0.318987
Pb	-1.034254	2.773970	-1.386675
Pb	-0.397950	2.448579	1.854701
Pb	-1.466294	-2.397897	-0.106317
Pb	-0.663478	-0.752772	2.648354
Pb	-2.748301	0.606246	0.429241
Cu	0.242608	0.112527	0.029282
Cu	0.998386	0.867974	-2.247722
Cu	-0.248673	1.886740	-4.055092
C	-0.656437	2.680446	-5.762039
C	-1.183345	1.940614	-6.855684
C	-0.388604	4.048083	-6.044100
C	-1.420662	2.506101	-8.116350
C	-0.620176	4.629383	-7.298285
C	-1.139481	3.859360	-8.348222
H	-1.828984	1.889714	-8.923633
H	-0.396152	5.688703	-7.459891
H	0.020208	4.687640	-5.252734
H	-1.418413	0.878229	-6.720532
H	-1.321863	4.305785	-9.328717

Table S47: $[PhAg_3Pb_{11}]^{3-}$, E = -122.723 eV

Pb	-0.223453	-2.974029	1.550357
Pb	2.110720	-2.382080	-0.726091
Pb	-2.096544	0.635953	-1.980671
Pb	-1.078788	-2.471786	-1.590869
Pb	2.366018	-0.938731	2.242931
Pb	3.055376	0.776579	-0.487745
Pb	0.474467	2.666172	-1.285094
Pb	1.316055	2.215048	1.910294
Pb	-2.855849	-1.064838	0.797182
Pb	-0.741881	-0.090987	3.200877
Pb	-1.906816	2.151412	0.968073
Ag	0.069331	-0.130255	0.289091
Ag	0.862834	-0.213327	-2.423831
Ag	-0.067242	1.620502	-4.252374
C	-0.477139	2.754100	-6.033269
C	0.551636	3.307004	-6.834580
C	-1.793149	2.971651	-6.510100
C	0.296310	4.021699	-8.015035
C	-2.068339	3.682467	-7.688595
C	-1.020504	4.213493	-8.450193
H	1.127935	4.430258	-8.597640
H	-3.104069	3.823345	-8.013119
H	-2.645121	2.574988	-5.946081
H	1.598664	3.179254	-6.535530
H	-1.226860	4.768888	-9.368416

Table S48: $[PhAu_3Pb_{11}]^{3-}$, E = 125.371 eV

Pb	-0.103894	-2.378843	1.906121
Pb	1.900297	-2.362750	-0.751524
Pb	-1.795670	1.363155	-1.618786
Pb	-1.334051	-1.859967	-1.128938
Pb	2.906218	-0.879048	2.050678
Pb	3.469399	0.528463	-0.899476
Pb	1.204927	2.859699	-1.480240
Pb	2.442150	2.398277	1.609008
Pb	-2.404754	-0.015822	1.384150
Pb	0.206277	0.634797	3.403028
Pb	-0.840967	2.946924	1.144657
Au	0.520162	0.284464	0.475327
Au	0.822920	-0.044216	-2.345444
Au	0.070461	1.648256	-4.316621
C	-0.414180	2.714040	-6.039268
C	0.575128	3.288253	-6.874616
C	-1.754119	2.879998	-6.468032
C	0.255016	3.978991	-8.051649
C	-2.086090	3.568693	-7.643020
C	-1.081077	4.123554	-8.444709
H	1.053302	4.406715	-8.665402
H	-3.135561	3.673288	-7.934062
H	-2.567714	2.460752	-5.867609
H	1.631306	3.194685	-6.602420
H	-1.335321	4.660631	-9.361502
